# Cancer-associated fibroblast driven paracrine IL-6/STAT3 signaling promotes migration and dissemination in invasive lobular carcinoma

**DOI:** 10.1186/s13058-025-02074-x

**Published:** 2025-07-01

**Authors:** Esme Bullock, Aleksandra Rozyczko, Sana Shabbir, Ifigenia Tsoupi, Adelaide I.J. Young, Jana Travnickova, Laura Gómez-Cuadrado, Zeanap Mabruk, Giovana Carrasco, Elizabeth Morrow, Kathryn Pennel, Pim Kloosterman, Julia M Houthuijzen, Jos Jonkers, Lidia Avalle, Valeria Poli, Richard Iggo, Xue Xiao, Jingjing Guo, Xuan Zhu, Elizabeth Mallon, Joanne Edwards, E. Elizabeth Patton, Valerie G. Brunton

**Affiliations:** 1 Cancer Research UK Scotland Centre (Edinburgh), Institute of Genetics and Cancer, University of Edinburgh, Crewe Road South, Edinburgh, EH4 2XR UK; 2 MRC Human Genetics Unit, Institute of Genetics and Cancer, University of Edinburgh, Crewe Road South, Edinburgh, EH4 2XR UK; 3 School of Cancer Sciences, Wolfson Wohl Cancer Research Centre, University of Glasgow, Glasgow, G61 1BD UK; 4 Division of Molecular Pathology, Oncode Institute, The Netherlands Cancer Institute, Amsterdam, The Netherlands; 5 Molecular Biotechnology Center, Department of Molecular Biotechnology and Health Sciences, University of Turin, Via Nizza 52, Turin, 10126 Italy; 6 INSERM U1218, Institut Bergonié, University of Bordeaux, Bordeaux, France; 7 Department of Pathology, School of Medicine, Sichuan Provincial People’s Hospital, University of Electronic Science and Technology of China, Chengdu, China; 8 Department of Pathology, Queen Elizabeth University Hospital, Glasgow, UK; 9 Present Address: International Research Center in Critical Raw Materials for Advanced Industrial Technologies (ICCRAM), R&D Center, Universidad de Burgos, Plaza de Misael Bañuelos s/n, Burgos, 09001 Spain

## Abstract

**Background:**

Invasive lobular carcinoma (ILC) is the second most common histological subtype of breast cancer after invasive ductal carcinoma of no special type (NST), accounting for 10–15% of diagnoses. Despite the myriad molecular, histological and clinical differences between ILC and NST tumors, patients are treated in the same way, and although prognosis initially is good, ILC patients have poorer long-term outcomes. Understanding the differences between these two subtypes and identifying ILC-enriched therapeutic targets is necessary to improve patient care.

**Methods:**

Human and mouse cancer-associated fibroblasts (CAFs), ILC cell lines and patient-derived organoids were used for in vitro and in vivo studies, including western blotting, migration, organotypic invasion assays and dissemination in zebrafish embryos. RNASeq was used to identify CAF and interleukin-6 (IL-6)-derived gene signatures. Bioinformatic analysis of public databases and immunohistochemical of human tumor microarrays was carried out.

**Results:**

We identified IL-6 as a paracrine CAF-derived factor that activates Signal-Transducer-and-Activator-of-Transcription-3 (STAT3) in human and mouse ILC models. Analysis of human breast tumors showed that the IL-6/JAK/STAT3 pathway is enriched in ER + ILC compared to ER + NST. A 42-gene CAF dependent IL-6 gene signature and 64-gene consensus IL-6 gene signature were generated and were significantly enriched in ER + ILC, with many of the genes overexpressed in ILC tumors. IL-6 treatment suppressed downstream estrogen signaling and also led to the acquisition of a more mesenchymal-like phenotype associated with increased migration and invasion. Finally, IL-6 treatment significantly increased ILC cell dissemination following injection into zebrafish embryos.

**Conclusions:**

CAF-derived IL-6 drives paracrine activation of the IL6/JAK/STAT3 signaling pathway which is enriched in ILC. This leads to the acquisition of pro-tumorigenic phenotypes, highlighting the pathway as a potential therapeutic target in ILC.

**Supplementary Information:**

The online version contains supplementary material available at 10.1186/s13058-025-02074-x.

## Introduction

Invasive lobular breast cancer (ILC) is the second most common histological subtype of breast cancer after invasive breast cancer of no special type (NST), commonly referred to as invasive ductal carcinoma. It accounts for around 15% of breast cancer cases with more than 90% expressing the estrogen receptor (ER). ILC has several distinguishing molecular and clinical features (reviewed in [1]): most ILC tumors have inactivating mutations in *CDH1*, encoding for the adherens junction protein E-cadherin, which contributes to the characteristic single file invasive growth pattern. ILC also has a distinct pattern of metastatic spread and often relapses later than NST contributing to poorer long-term outcomes for patients [2, 3]. Several studies have profiled the genomic landscape in ILC and also found differences in the frequency of mutations in common cancer genes compared to NST [4–7]. But despite these differences, the histological ILC subtype is not considered when planning treatments for patients with ILC, and further studies are required to understand the unique biology of ILC.

Cancer-associated fibroblasts (CAFs) are the main stromal cell type in the tumor microenvironment and are principally involved in extracellular matrix production and remodeling, in addition to secretion of factors that control tumor cell survival, metastatic spread and the immune environment [8, 9]. ILC is characterized by having a dense stromal content, which can in part be attributed to the single file invasive growth pattern, and differences in collagen deposition and alignment have been described between ILC and NST [10, 11]. Increased CAFs in ILC compared to matched NST samples has also been reported [12], while in a mouse model of ILC (mILC) (*WapCre; Cdh1*^*F/F*^;*Pten*^*F/F*^, WEPtn) there are increased numbers of CAFs compared to triple negative breast cancer models [13]. Single cell transcriptomic analysis of the WEPtn tumors identified distinct CAF subtypes which are found in human ILC CAFs [13], while in human tumors, differences in CAFs based on immunohistochemical markers were identified between ILC and NST [14, 15], and between different subtypes of ILC [16]. Additionally, we have characterized the molecular differences in CAFs from ER + ILC and ER + NST, identifying changes in matrix organization and growth factor signaling pathways [17]. However, it is not known how CAFs regulate the behavior of ILC tumor cells.

Here we asked how CAF driven paracrine signaling impacts on ILC and identified an interleukin-6 (IL-6)/Signal-Transducer-and-Activator-of-Transcription-3 (STAT3) signaling pathway that is enriched in ILC compared to NST. IL-6 secreted from CAFs suppresses estrogen signaling, induces a more motile mesenchymal-like phenotype, and promotes dissemination of ILC cells in zebrafish embryos.

## Results

### IL-6 secreted from CAFs activates STAT3 in ILC cells

**Fig. 1:**
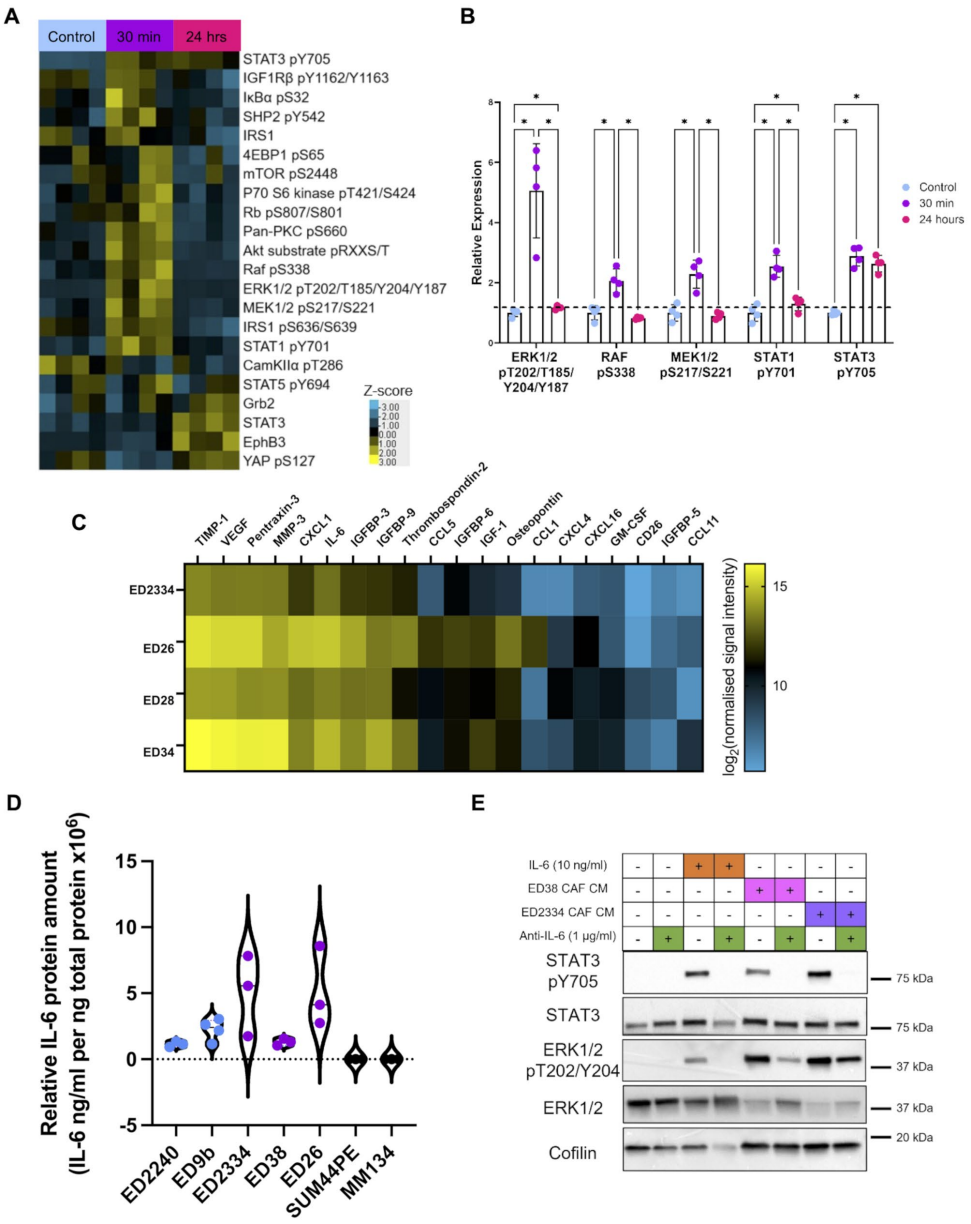
ILC CAF conditioned media drives STAT3 and MAPK pathway activation. (**A**) Heatmap of significantly changed proteins and phospho-proteins after 30 min–24 h of ED28 CAF conditioned media (CM) stimulation of SUM44PE cells compared to control cells. Protein expression was normalized to fast green stain for total protein and the heatmap shows the z-scores of expression. (**B**) Proteins with more than 2-fold and significant change in expression in the RPPA, determined by normalizing all values to mean of control. For (**A**) and (**B**), significance (* FDR < 0.05) was determined by two-way ANOVA, with multiple comparison correction by two-stage linear step-up procedure of Benjamini, Kreiger and Yeketel (BKY) in GraphPad Prism. (**C**) Heatmap of the top 20 most highly secreted proteins by primary ILC patient derived CAFs, *n* = 3 biological replicates for ED2334, ED26 and ED28, *n* = 2 for ED34. Heatmap shows log_2_ of signal intensity normalized to CAF cell pellet protein concentration, showing the mean across replicates. (**D**) Concentration of IL-6 in CM collected after 72 h from primary NST CAFs (blue), ILC CAFs (purple), and the ILC tumor cell lines SUM44PE and MM134 determined by ELISA, normalized to cell pellet protein concentration. (**E**) Western blot of SUM44PE cells stimulated for 30 min with recombinant human IL-6 (10 ng/mL) or CAF-CM from primary ILC CAFs (ED38 and ED2334) +/- anti-IL-6 blocking antibody (1 µg/mL)

SUM44PE ILC cells were treated with conditioned media (CM) collected from CAFs isolated from ER + ILC tumors [17], resulting in activation of a number of signaling pathways (Fig. 1A). Most notably, phosphorylated markers of the STAT1, STAT3 and MAPK pathways had a more than two-fold significant increase in expression following CAF-CM stimulation, with activation of STAT3 being maintained over 24 h (Fig. 1B). This was associated with an increase in total STAT3 after 24 h, consistent with a known positive feed-forward loop where STAT3 activation increases its own transcription [18]. Phosphorylation of STAT3 and the increase in total STAT3 expression were validated by western blot (Supplementary Fig. S1A).

A forward phase array identified a number of chemokines and cytokines secreted by ILC patient-derived CAFs, as well as components of the IGF-1 signaling pathway, a pathway known to be hyperactivated in ILC tumors (Fig. 1C) [19, 20]. One of the most highly expressed proteins present in the CM from the CAFs was the cytokine IL-6. IL-6 drives a wide range of pro-tumorigenic phenotypes and is a well-known activator of STAT1, STAT3 and MAPK pathways [21]. IL-6 secretion by ILC CAFs was confirmed by ELISA and was detected at levels equivalent to those required for STAT3 phosphorylation following treatment with recombinant IL-6 (Fig. 1D and Supplementary Fig. S1B). IL-6 was also secreted by CAFs isolated from ER + NST tumors, however, the ILC cell lines SUM44PE and MDA-MB-134VI (MM134) did not secrete detectable levels of IL-6 (Fig. 1D). In addition, expression of *IL6* was undetectable in RNA-Seq analysis of SUM44PE and MM134 ILC cell lines and two ILC patient-derived organoid (PDO) models (HCI-013 and − 018 [22]). Analysis of single cell RNAseq (scRNAseq) from WEPtn mILC tumors showed that *Il6* expression is restricted to the stromal compartment, with the highest expression seen in CAF populations (Supplementary Figure S1C), which was confirmed by ELISA in CM from cells isolated from the WEPtn mILC model (Supplementary Fig. S1D) [13]. Further analysis of two ER + human ILC tumors demonstrated stromal expression of *IL6*, with highest expression seen in CAFs, perivascular-like cells and endothelial cells with relatively low levels in the epithelial cancer cells (Supplementary Figure S1E and F) [23]. Together, this supports the presence of a paracrine signaling IL-6/STAT3 pathway in human and mouse ILC.

Treatment of SUM44PE cells and MM134 ILC cells with CM from ILC CAF lines for 30 min resulted in phosphorylation of STAT3 and ERK1/2. Addition of an IL-6 neutralizing antibody to the CAF-CM completely abrogated STAT3 Y705 phosphorylation and reduced ERK1/2 phosphorylation, demonstrating that IL-6 in CAF-CM is responsible for activation of STAT3 (Fig. 1E and Supplementary Fig. S1G). Similar results were seen in the mILC model with CM-induced stimulation of STAT3 Y705 phosphorylation being blocked following treatment with the anti-IL-6 neutralizing antibody (Supplementary Fig. S1H). Phosphorylation of STAT3 downstream of IL-6 can be mediated via Janus kinase (JAK) and treatment with the JAK inhibitor baricitinib also prevented IL-6 and CM-dependent STAT3 Y705 phosphorylation (Supplementary Fig. S1I). Phosphorylation of STAT3 induces its translocation to the nucleus where it acts as a transcription factor to regulate the expression of multiple genes, and treatment of SUM44PE cells with IL-6 resulted in increased expression of nuclear STAT3 (Supplementary Fig. S1J).

### IL-6/JAK/STAT3 pathway is enriched in ER + ILC tumors

**Fig. 2:**
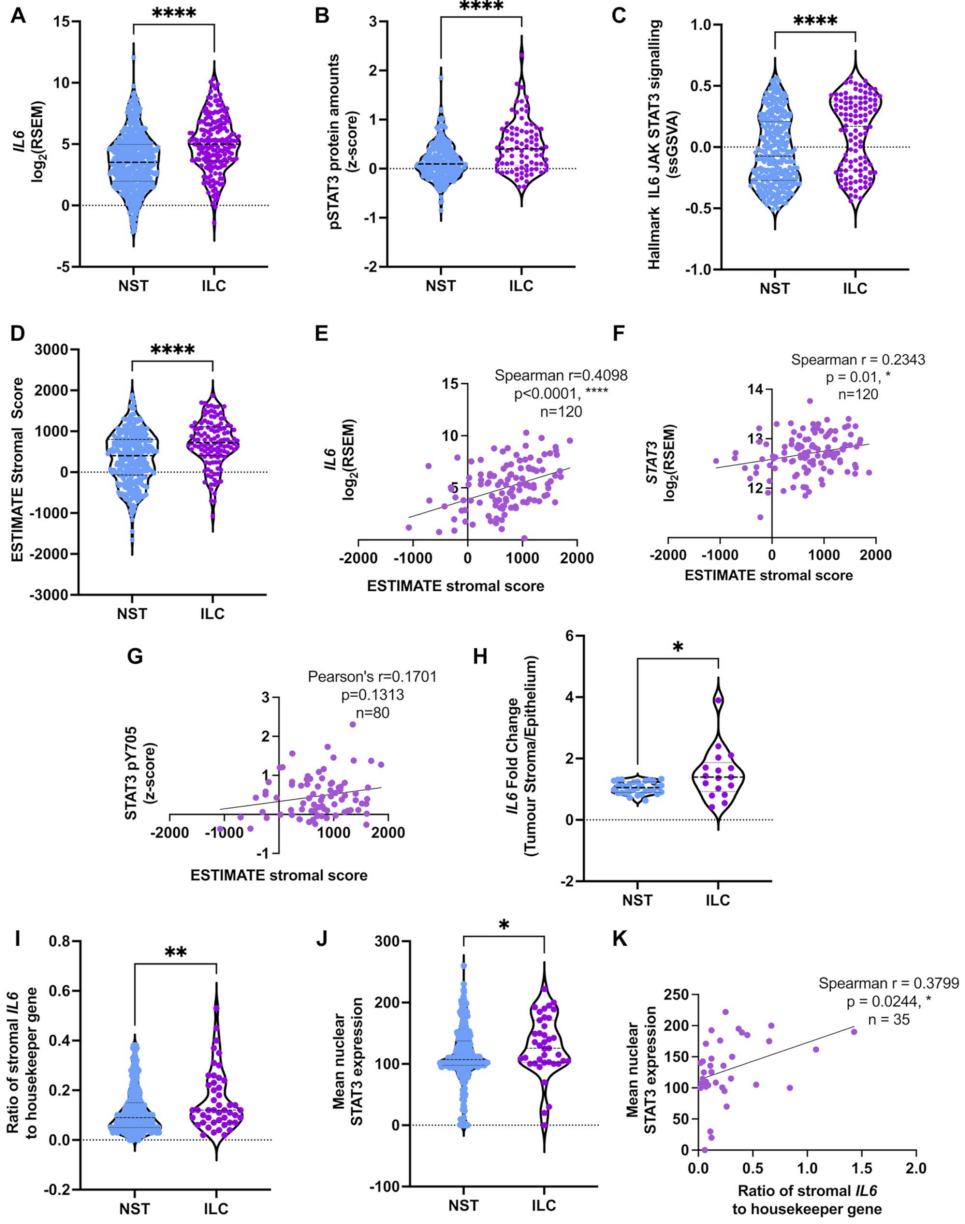
IL-6/STAT3 pathway is augmented in ER + ILC compared to ER + NST tumors. (**A**, **B**) Expression of *IL6* (**A**) and pSTAT3 (**B**) in TCGA RNAseq and RPPA dataset, ER + ILC (*n* = 191) and ER + NST (*n* = 555). (**C**) Enrichment of “Hallmark IL6 JAK STAT3 signaling” in ER + ILC following GSVA of TCGA RNA-Seq dataset. (**D**) ESTIMATE stromal scores (NST *n* = 332, ILC *n* = 120). **E-G** Spearman correlation of ESTIMATE stromal score with *IL6* (**E**), *STAT3* (**F**) and pSTAT3 (**G**) expression in ER + ILC tumors (RNAseq data *n* = 120, RPPA data *n* = 80). (**H**) Fold change of *IL6* expression in stroma compared to tumor epithelial compartment in laser capture microdissection data for NST (*n* = 34, GSE68744) and ILC (*n* = 17, GSE148398). (**I**) Expression of *IL6* in the stromal compartment of ER + NST (*n* = 412) and ER + ILC (*n* = 63) determined by RNAscope, *p* = 0.008. (**J**) Expression of nuclear STAT3 in the tumor compartment of ER + tumors (NST *n* = 332, ILC *n* = 39), *p* = 0.019. Unpaired two-tailed (**A** and **D**) t-test and (**B**, **C**, **H**) Mann-Whitney tests in GraphPad Prism, * *p* < 0.05, ** *p* < 0.01, **** *p* < 0.0001. (**I-J**) two-tailed Mann-Whitney U tests in SPSS. **K** Spearman correlation between stromal *IL6* and tumoral nuclear STAT3 in ER + ILC tumors (*n* = 35). All correlation analysis carried out in GraphPad Prism

To understand the relevance of the IL-6/JAK/STAT3 pathway to human ILC and whether the same pathway is active in NST, analysis of ER + ILC and ER + NST tumors in the Cancer Genome Atlas (TCGA, cbioportal.org [24]) was carried out. ER + ILC tumors have significantly higher expression of *IL6* and STAT3 pY705 than ER + NST tumors (Fig. 2A, B). Additionally, single sample gene set variation analysis (GSVA) revealed a significant enrichment of the Hallmark gene set “IL6-JAK-STAT3 signaling” in ER + ILC tumors compared to ER + NST tumors (Fig. 2C).

As we have shown that IL-6 is secreted from CAFs, the increase in *IL6* may reflect an increase in the stromal content of ILC tumors. Using the ESTIMATE stromal score [25] we confirmed that the stromal content was significantly higher in ER + ILC compared to ER + NST tumors in TCGA dataset (Fig. 2D), and that this positively correlated with *IL6* and *STAT3* expression, with a similar trend seen for STAT3 pY705 (Fig. 2E-G). Analysis of datasets where tumor stroma was separated from tumor epithelium [17, 26], showed that in some ER + ILC tumors there was an increase in stromal expression of *IL6* relative to the tumor epithelium compared to ER + NST tumors (Fig. 2H) suggesting that *IL6* may be more highly expressed by the stromal compartment of ILC than NST tumors. To address this directly, stromal *IL6* expression was determined by RNAscope in a retrospective cohort of 538 ER + NST and ILC tumors. Analysis of *IL6* transcripts per µm^2^ of stroma in each tissue core showed a significant increase in *IL6* in the stroma of ER + ILC compared to ER + NST tumors (Fig. 2I and Supplementary Figure S2A) indicating that in addition to the increased stromal content of ILC tumors, an increased stromal expression of *IL6* may also contribute. IL-6 receptor immunohistochemistry was carried out on the same cohort of tumors and showed that there was no difference in expression between ER + NST and ILC tumors (Supplementary Figure S2B). However, as a marker of pathway activation, there was an increase in nuclear tumoral STAT3, with a significant positive correlation between *IL6* and nuclear STAT3 (Fig. 2J, K). The increase in nuclear tumoral STAT3 was not confirmed in an additional cohort of ER + NST and ILC tumors. The reason for this discrepancy is not known, but in this cohort a small number of ILC tumors had very low levels of nuclear STAT3 (Supplementary Figure S2C). Due to the small numbers of ILC patients in the Glasgow and Chengdu cohorts, the impact of STAT3 on survival in ILC was analyzed in a larger cohort of ILC tumors (Supplementary Figure S2D). When STAT3 expression was scored on subcellular localization, high cytoplasmic STAT3 was associated with worse survival (Supplementary Figure S2E), and was an independent variable on multivariate Cox regression analysis (Supplementary Table S1), but there was no association between nuclear STAT3 expression and survival (Supplementary Figure S2F). This is consistent with our previous study in a cohort of 527 breast cancer patients which showed that high expression of cytoplasmic STAT3 was associated with reduced outcome while low expression of nuclear STAT3 was associated with poor outcomes [27]. It is not clear what the relevance of cytoplasmic STAT3 is and previous studies looking at nuclear STAT3 expression and survival in breast cancer patients have reported conflicting results describing an association of increased expression with either improved or worse survival (reviewed in [28]). In one study pSTAT3 was associated with poor outcome only when focusing specifically on patients with IL6 positive tumors [29], suggesting that as STAT3 has pleotropic effects and can be activated by numerous pathways a more targeted approach would yield more meaningful data. Therefore, further survival analysis was carried out on the Hallmark gene set “IL6-JAK-STAT3 signaling” and also our CAF induced IL-6 gene signature (CAF-IL6GS) (see below) in ER + ILC tumors and ER + NST tumors in TCGA (Supplementary Figure S3A-D). This showed that both overall survival (OS) and progression-free survival (PFS) were associated with high levels of pathway activation in ER + NST while in ER + ILC high levels of pathway activation were associated with worse survival although this did not always reach significance (Supplementary Figure S3) but are indicative of subtype specific effects. In the RATHER cohort of 79 ER + ILC patients with associated survival data [6], Hallmark IL6-JAK-STAT3 signaling was associated with better breast cancer specific survival. However, our CAF-IL6GS was significantly associated with poorer recurrence free survival in ER + ILC patients (Supplementary Figure S3E and F).

### IL-6 secreted from CAFs drives gene expression in ILC cells

**Fig. 3:**
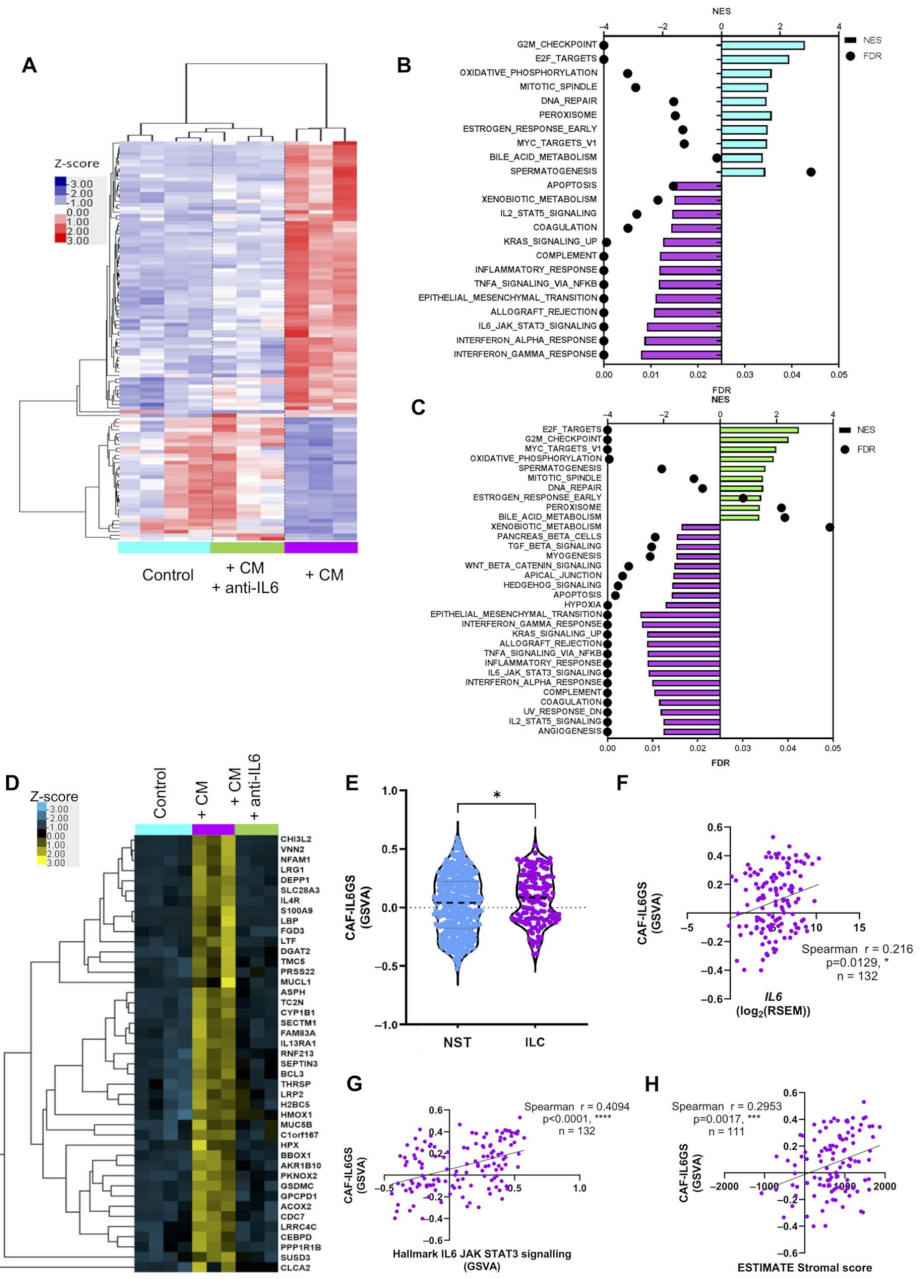
IL-6 in CAF conditioned media drives gene expression in ILC cells. (**A**) SUM44PE ILC cells were stimulated for 24 h with CAF-CM from ED26 ILC CAFs +/- IL-6 neutralizing antibody (1 µg/mL). Heatmap showing unbiased hierarchical clustering of the 110 differentially expressed genes determined by a generalized linear model likelihood ratio ANOVA-like test, FDR < 0.05. (**B**) Significantly enriched Hallmark gene sets in control (blue) and conditioned media treated samples (purple), FDR < 0.05 and, (**C**) CAF-CM stimulated cells (purple bars) were compared to CAF-CM + anti-IL-6 stimulated cells (green bars), FDR < 0.05. (**D**) Heatmap of the 43 genes in the CAF induced IL-6-dependent gene signature (CAF-IL6GS) from the RNA-Seq dataset. (**E**) CAF-IL6GS score calculated by ssGSVA in ER + NST (*n* = 427) and ER + ILC (*n* = 132) tumors in TCGA, Mann-Whitney test *p* = 0.0154 using GraphPad Prism. CAF-IL6GS score positively and significantly correlates with, (**F**) *IL6* expression, (**G**) Hallmark IL6-JAK-STAT3 gene signature (*n* = 132) and, (**H**) ESTIMATE stromal score (*n* = 111) in ER + ILC tumors, Spearman correlation, * *p* < 0.05, *** *p* < 0.001, **** *p* < 0.0001 carried out using GraphPad Prism

To understand the downstream effects of IL-6/STAT3 pathway activation on gene expression, we carried out RNA-Seq on SUM44PE cells stimulated with CM collected from ILC CAFs in the presence or absence of an IL-6 neutralizing antibody to block STAT3 activation (Supplementary Fig. S4A). Differential gene expression analysis identified 110 differentially expressed genes between the three groups, with the majority being driven by IL-6 within the CAF-CM (Fig. 3A). Gene set enrichment analysis (GSEA [30, 31]), showed that CAF-CM treated cells were significantly enriched for a number of signaling pathways, including IL6-JAK-STAT3, epithelial-to-mesenchymal transition (EMT) and apoptosis gene sets, relative to control cells (Fig. 3B) and cells treated with CAF CM + anti-IL-6 antibody (Fig. 3C) consistent with activation of the STAT3 pathway which is known to induce EMT and induce expression of genes associated with the control of apoptosis. In contrast, control cells and cells treated with CAF CM + anti-IL-6 antibody both were significantly enriched for proliferative gene sets. Expression of some of the most highly induced genes was validated by RT-qPCR and were shown to be IL-6 dependent (Supplementary Fig. S4B).

To determine if expression of the IL-6-dependent genes required STAT3 activity downstream of IL-6, SUM44PE cells were treated with siRNA against luciferase (siCTRL) or STAT3 (siSTAT3) [32]. siSTAT3 treatment effectively inhibited expression of *STAT3* and completely abolished the IL-6 dependent increase in *STAT3* expression (Supplementary Fig. S4C). While some genes were STAT3-dependent (e.g. *MUCL1*, *SERPINB5*, *BCL3*, *LRG1*) there were genes whose expression was not completely dependent on STAT3 (e.g. *S100A8*).

Addition of the IL-6 neutralizing antibody to CAF-CM identified 43 genes whose expression was significantly upregulated by CAF CM in an IL-6 dependent manner (> 2-fold increase in expression CAF-CM versus control and significantly reduced with the addition of the IL-6 neutralizing antibody) (Fig. 3D and Supplementary File S1 and S2). A score of this CAF induced IL-6 gene signature (CAF-IL6GS) was calculated for individual ER + tumors in TCGA dataset and was significantly more highly expressed in ER + ILC tumors than NST tumors (Fig. 3E). 29/43 of the genes were differentially expressed between ER + ILC and NST tumors in the TCGA RNA-Seq dataset, with 23 of these genes being significantly more highly expressed in ILC tumors (Supplementary Fig. S4D). There was a significant positive correlation of the CAF-IL6GS with *IL6* expression in ER + ILC tumors (Fig. 3F) and the “Hallmark IL6-JAK-STAT3 signaling” gene set (Fig. 3G). The CAF-IL6GS score significantly correlated with the ESTIMATE stromal score in ILC tumors (Fig. 3H) consistent with a positive correlation between *IL6* and ESTIMATE stromal score in these tumors (Fig. 2E).

**Fig. 4:**
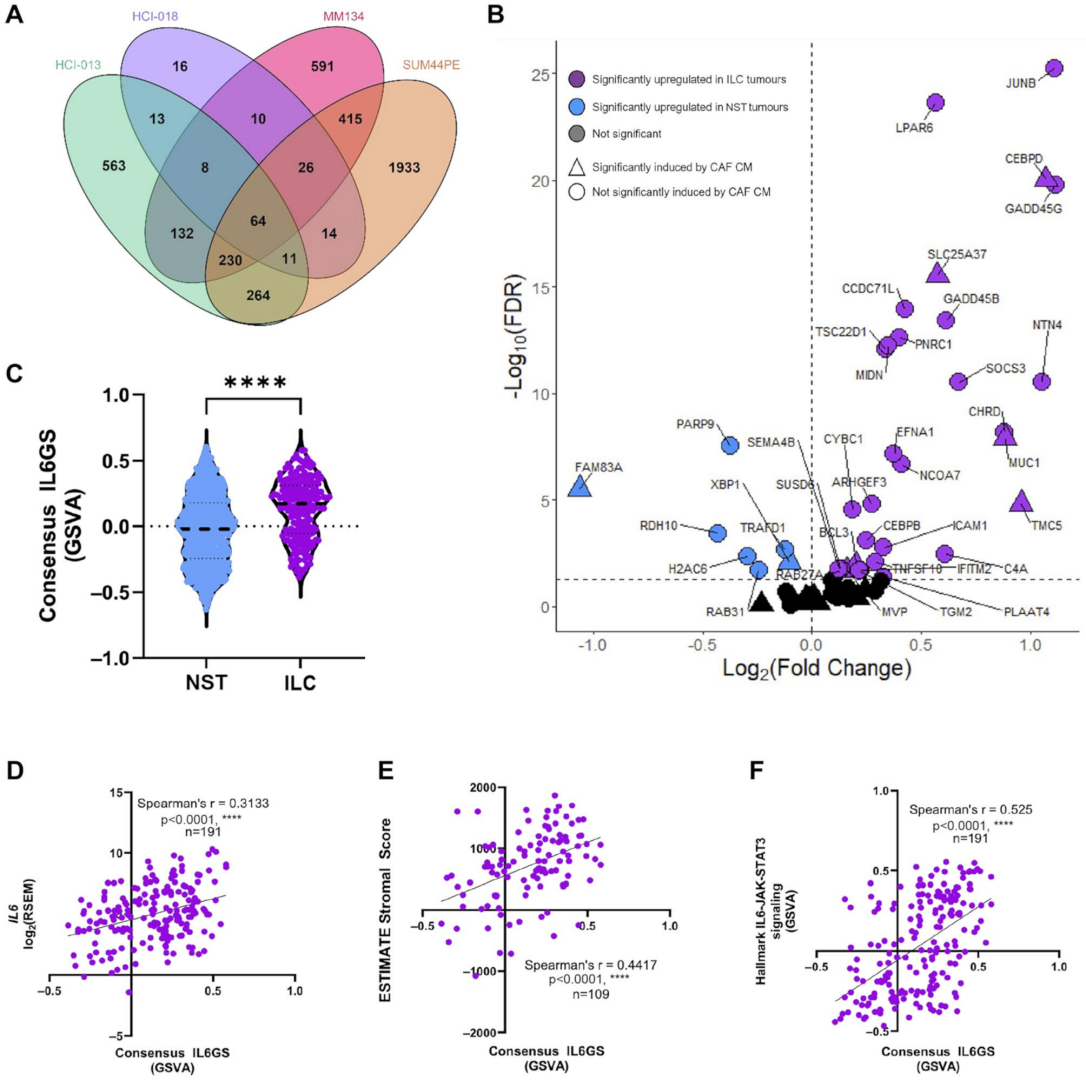
IL-6 drives consistent gene changes in multiple ILC models. (**A**) Venn diagram of differentially expressed genes in IL-6 treated HCI-013 (green), HCI-018 (purple) ER + ILC PDOs and MM134 (pink) and SUM44PE (orange) ILC cell lines. 64 genes were differentially expressed by IL-6 in all four models. (**B**) Volcano plot of differential expression of 61 genes in the consensus IL6GS between ER + ILC (*n* = 191) and ER + NST (*n* = 563) tumors in the TCGA Firehose dataset. Genes significantly differentially expressed (Wilcoxon U test, FDR < 0.05) and upregulated in ILC (purple) or NST (blue), triangles indicate genes also significantly upregulated by CAF CM in SUM44PE cells. (**C**) Consensus IL6GS score calculated by GSVA in ER + ILC and ER + NST tumors from TCGA, Mann-Whitney test **** *p* < 0.0001 in GraphPad Prism. Expression of the Consensus IL6GS score significantly and positively correlates with (**D**) *IL6*, (**E**) ESTIMATE stromal score (*n* = 109), and (**F**) Hallmark IL6-JAK-STAT3 signaling gene set (*n* = 191) in ER + ILC tumors, Spearman correlation in GraphPad Prism

RNA-Seq analysis of IL-6 dependent changes in gene expression in ILC cell lines (SUM44PE, MM134) and ER + ILC PDO models (HCI-013, HCI-018) identified 64 differentially expressed genes that were consistently altered across all models (Fig. 4A, Supplementary Fig. S5A-E and Supplementary File S1), referred to as the ‘consensus IL6 gene signature (IL6GS)’. Expression of some of the most highly differentially expressed genes were validated by qPCR (Supplementary Fig. S5F-H), with the same trends seen in an additional ILC PDO, LA-PDxO [33]. 15 genes in the consensus IL6GS were also significantly upregulated by CAF CM in an IL-6 dependent manner (Supplementary Fig. S5I), and all but 10 of the genes significantly differentially regulated by CAF CM in SUM44PE cells were also significantly differentially regulated by IL-6 stimulation (Supplementary Fig. S5J). 30/38 of the differentially expressed consensus IL6GS genes between ILC and NST tumors in the TCGA dataset were upregulated in ILC tumors (Fig. 4B). Expression of the consensus IL6GS was significantly enriched in ER + ILC tumors compared to ER + NST tumors (Fig. 4C) and significantly positively correlated with *IL6* expression (Fig. 4D) and the ESTIMATE stromal score in ILC tumors (Fig. 4E). There was also a significant positive correlation with the “Hallmark IL6-JAK-STAT3 signaling” gene set (Fig. 4F).

### Effects on proliferation of ILC cells

To determine if the enrichment of proliferative gene sets in SUM44PE cells treated with CAF-CM and anti-IL-6 blocking antibody was associated with suppression of proliferation by IL-6, SUM44PE cells were stimulated with recombinant human IL-6. Using incorporation of the thymidine analog 5’-ethynyl-2’-deoxyuridine (EdU) into the DNA of cells that transit through S-phase showed a small but non-significant decrease in the number of EdU + SUM44PE cells following IL-6 treatment (Supplementary Fig. S6A), and a small but significant reduction in proliferation of WEPtn mILC cell lines (Supplementary Fig. S6B, C).

### IL-6 suppresses Estrogen receptor signaling

**Fig. 5:**
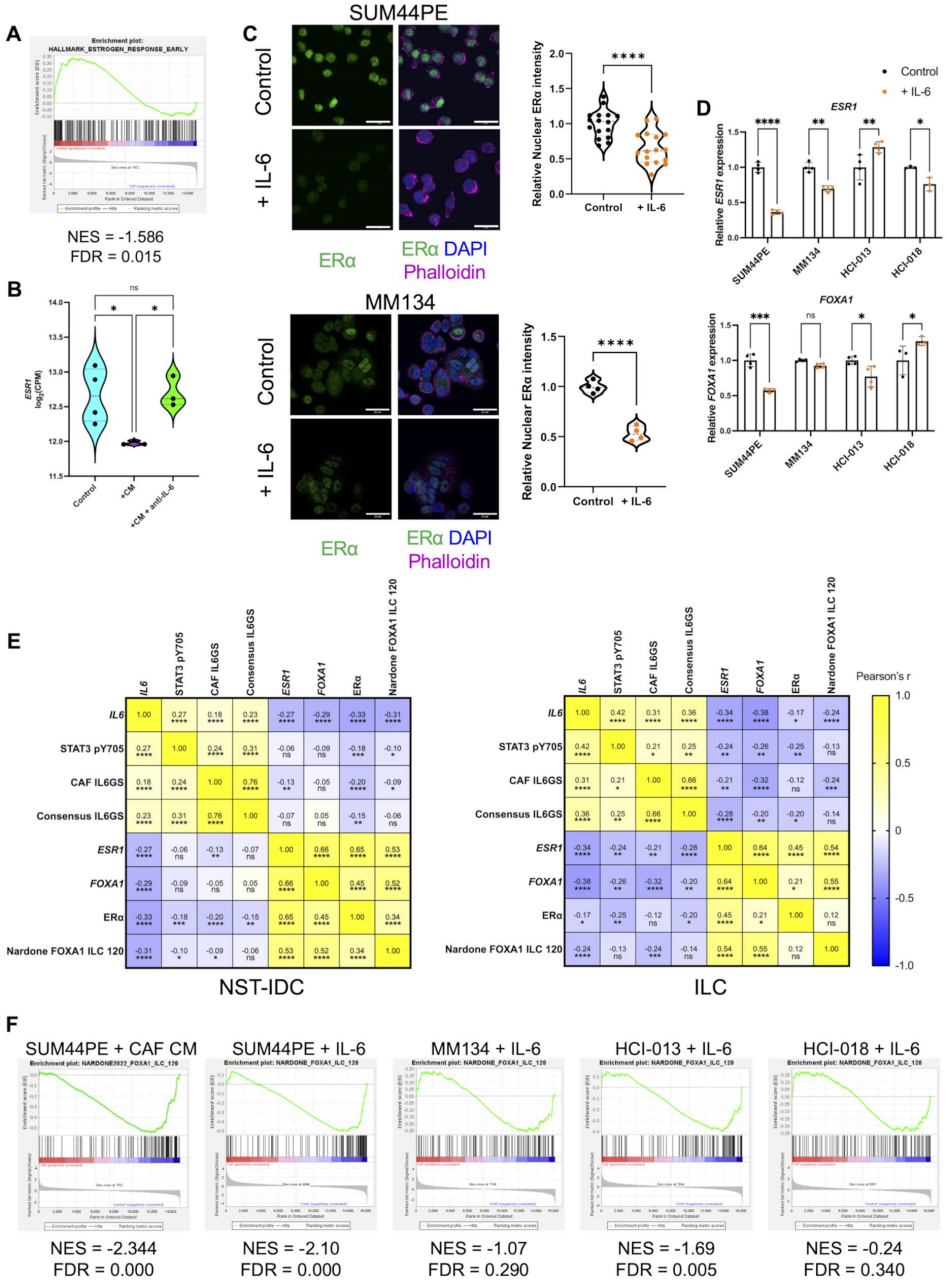
IL-6 regulates estrogen receptor and FOXA1 expression in ILC tumor cells. (**A**) GSEA plot of the Hallmark Estrogen Response Early gene set comparing control SUM44PE cells to CAF CM stimulated cells. (**B**) Expression of *ESR1* in SUM44PE cells stimulated with CAF CM +/- anti-IL-6. (**C**) Representative images of (top) SUM44PE and (bottom) MM134 cells cultured for 1 week in complete media +/- 10 ng/ml recombinant human IL-6, showing ERα expression (green), nuclei labelled with DAPI (blue) and actin with phalloidin (magenta). Images at 60X magnification, scale bar showing 25 μm. Quantification of relative nuclear localization (mean gray value normalized to mean of control cells) of ERα in SUM44PE cells (*n* = 3 biological replicates with 5–6 random fields of view (FoVs) per condition) and MM134 *n* = 4 random FoVs. Each point represents the mean relative intensity of one FoV normalized to the mean of the control. Unpaired t-test in GraphPad Prism, **** *p* < 0.0001. (**D**) Relative expression of (top) *ESR1* and (bottom) *FOXA1* in SUM44PE, MM134, HCI-013 and HCI-018 + IL-6, showing expression normalized to control cells, each point representing a biological replicate. Two-way ANOVA with Šídák’s multiple comparisons test in GraphPad Prism, adjusted * *p* < 0.05, ** *p* < 0.01, *** *p* < 0.001, **** *p* < 0.0001. (**E**) Correlation matrices of *IL6*, *ESR1*, *FOXA1* (RNAseq), STAT3 pY705, ER⍺ (RPPA), Nardone FOXA1 signature and CAF and consensus IL6GS (ssGSVA scores) in (left) ER + NST and (right) ER + ILC in the TCGA dataset. NST, RNAseq and ssGSVA scores *n* = 555, RPPA *n* = 420, ILC RNAseq and ssGSVA *n* = 191, RPPA *n* = 143. Values shown are Pearson’s r carried out in GraphPad Prism, not significant ns, * *p* < 0.05, ** *p* < 0.01, *** *p* < 0.001, **** *p* < 0.0001. (**F**) GSEA plots of enrichment of the ILC-specific 120-gene FOXA1 signature described by Nardone et al. (**A**) and (**F**), NES < 0 enriched in control cells, FDR < 0.05 considered significant

The majority of ILC patients are ER + and are therefore treated using endocrine targeted therapies. CAF-CM treatment of SUM44PE cells led to a significant suppression of Hallmark estrogen signaling gene sets in an IL-6 dependent manner (Figs. 3B and C and 5A). This was associated with a down-regulation of *ESR1* in the CAF-CM treated SUM44PE cells which was restored following treatment with IL-6 blocking antibody (Fig. 5B), and was validated at the protein level with decreased ERα expression in IL-6 stimulated SUM44PE and MM134 cells (Fig. 5C). However, there were differences in *ESR1* expression in response to IL-6 across the ILC models: IL-6 treatment only downregulated *ESR1* in SUM44PE, MM134 and HCI-018, while in SUM44PE, MM134 and HCI-013 there was a significant down regulation of the ERα pioneer transcription factor *FOXA1* (Fig. 5D). When this analysis was extended to include ER + ILC and NST tumors in TCGA it showed that there was a significant negative correlation between *IL6*, *ESR1*, *FOXA1* and ERa in both subtypes (Fig. 5E), suggesting that the link between IL-6 and ER signaling is not unique to ER + ILC. However, ERα and FOXA1 signaling has been shown to be distinct in ILC and contributes to increased tamoxifen resistance [34]. GSEA demonstrated that IL-6 suppresses expression of the ILC-specific FOXA1 signature in all cell lines (Fig. 5F).

### IL-6 promotes a mesenchymal-like phenotype in ILC cells

**Fig. 6:**
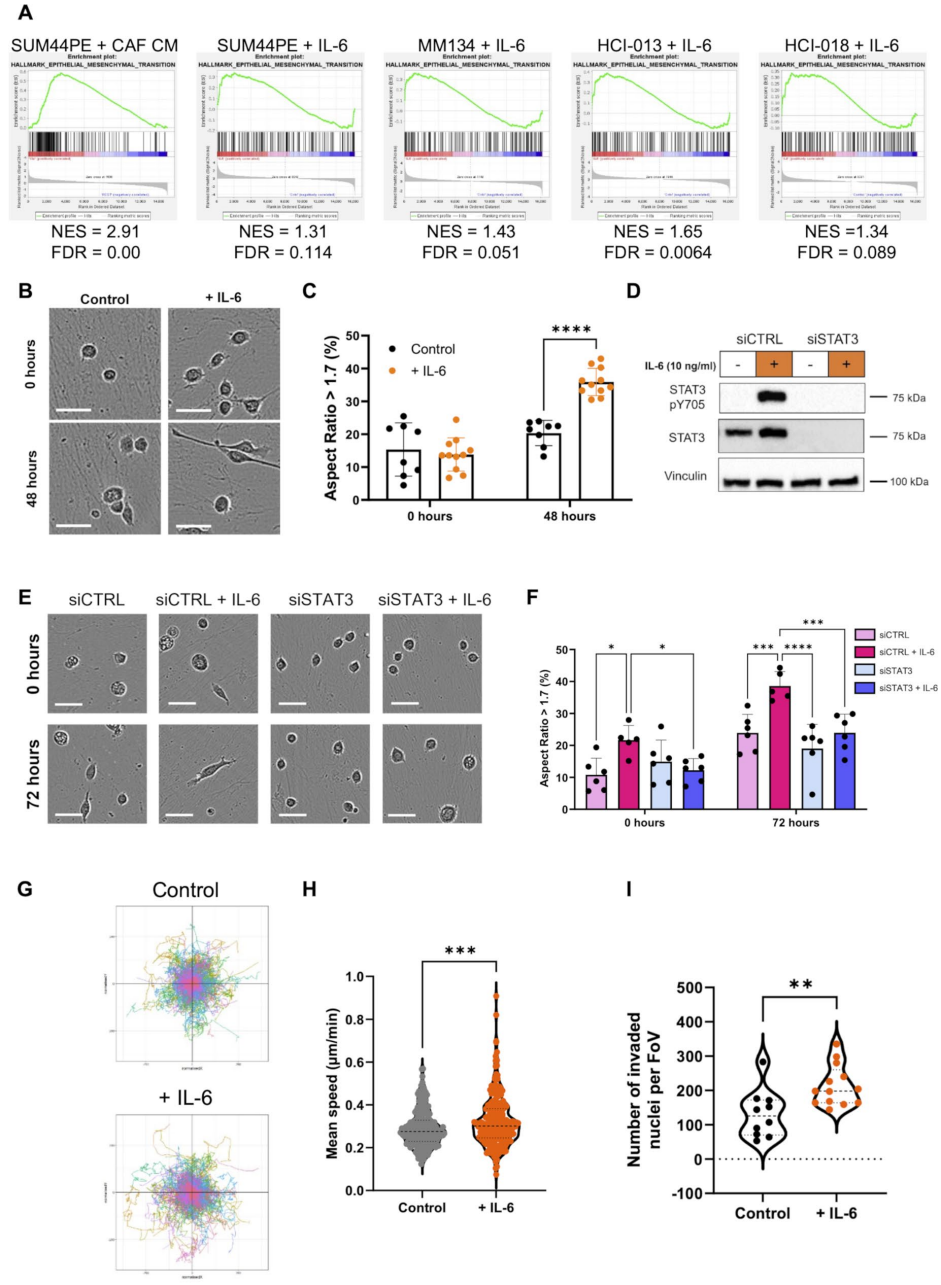
IL-6 drives a mesenchymal and pro-migratory phenotype in SUM44PE cells. (**A**) Enrichment of Hallmark EMT gene set in (left to right) CAF CM (NES > 0) treated SUM44PE cells compared to both control and CM + anti-IL6 cells, IL-6 treated SUM44PE, MM134, HCI-013 and HCI-018 compared to control. NES > 0, enriched in CAF CM or IL-6 treated cells, FDR < 0.05 considered significant. (**B**) Representative images of SUM44PE cells adhered to ILC CAF CDMs at time of stimulation (top, 0 h) and 48 h (bottom) after stimulation with IL-6 (10 ng/ml). (**C**) Quantification of the percentage of cells with AR > 1.7, indicating a mesenchymal phenotype. (**D**) Western blot of SUM44PE cells treated with siCTRL or siSTAT3 for 48 h then stimulated +/- IL-6 for 24 h. (**E**) Representative images of SUM44PE cells treated with siCTRL or siSTAT3 and seeded onto ILC CAF CDMs. Images at time of stimulation (top, 0 h) and (bottom) 72 h after IL-6 stimulation. (**F**) Quantification of percentage of cells with AR > 1.7. (**B**) and (**E**) – Images taken at 10x on Incucyte S3, scale bar shows 50 μm, (**C**) and (**F**) – *n* = 3 biological replicates with two FoVs per replicate, two-way ANOVA with (**C**) Sidak’s and (**E**) Tukey’s multiple comparison tests in GraphPad Prism * adjusted *p* < 0.05, ** adjusted *p* < 0.01, *** adjusted *p* < 0.001, **** adjusted *p* < 0.0001. (**G**) Representative co-ordinate plots of SUM44PE migration over 72 h +/- IL-6. (**H**) Speed of individual SUM44PE cells over 72 h in complete media +/- IL-6 (10 ng/ml), images taken every 30 min, *n* = 3 biological replicates, unpaired t-test *p* < 0.001. Analyzed using Trackmate plugin in ImageJ. (**I**) Quantification of number of SUM44PE cells that have undergone haptotaxis towards collagen. Number of nuclei, control *n* = 3 biological replicates, + IL-6 *n* = 4 biological replicates with 3 or 4 FoVs imaged per repeat, each point represents one FoV, Mann-Whitney test ** *p* < 0.01 in GraphPad Prism

GSEA identified a significant enrichment for EMT genes in the CAF-CM treated SUM44PE cells compared to both control and anti-IL-6 treated cells (Fig. 3B and C). The Hallmark EMT gene set [30, 31] was also enriched in IL-6 treated SUM44PE, MM134, HCI-013 and HCI-018 cells compared to controls (Fig. 6A). ILC cell lines display a rounded epithelial-like morphology, rather than a mesenchymal-like elongated phenotype, despite loss of E-cadherin expression, a key feature of EMT. However, when SUM44PE cells were seeded on ILC CAF-derived extracellular matrices (CDMs), an IL-6 dependent elongation and morphological change could be seen (Fig. 6B). Aspect ratio can be used to quantify morphological changes: a perfect circle has an aspect ratio of 1 and the more elongated a cell, the larger its aspect ratio, with mesenchymal-like cells previously having been defined as having an aspect ratio > 1.7 [35]. Addition of IL-6 significantly increased the percentage of cells with an aspect ratio > 1.7 (Fig. 6C). Treatment with siSTAT3, which prevented the IL-6-dependent expression of STAT3 (Fig. 6D), confirmed that induction of the mesenchymal-like morphology was dependent on STAT3 activity downstream of IL-6 stimulation (Fig. 6E, F).

### IL-6 promotes the migratory and invasive capacity of ILC cells

Stimulation of SUM44PE cells with IL-6 lead to a significant increase in random migration (Fig. 6G, H), and IL-6 pre-treatment also drove an increase in haptotaxis towards collagen I (Fig. 6I). Migration of the mouse 13-MCB-17 cells in a wound healing assay also increased following pre-treatment with IL-6 (Supplementary Fig. S7A, B). IL-6 has also been reported to be involved in tumor cell invasion, but the human ILC cell lines including SUM44PE have limited ability to invade using conventional assays [36]. However, invasion of the mILC cell lines using an organotypic invasion assay [37], showed a significantly higher invasion index for cells that had been pre-treated with IL-6 compared to untreated cells (Supplementary Fig. S7C, D). There was also an increase in STAT3 pY705 in the IL-6 pre-treated cells that had invaded into the matrices (Supplementary Fig. S7E).

### IL-6 promotes the dissemination of ILC cells in zebrafish embryos

To determine if IL-6 drives SUM44PE cell migration and dissemination in vivo, a zebrafish embryo xenograft assay was used [38]. Use of Casper *Tg(fli1*:eGFP) zebrafish embryos allowed visualization of DiI-labeled SUM44PE cells in the GFP-labeled vasculature following injection into the perivitelline space (PVS) two days post-fertilization (2dpf) (Supplementary Fig. S8A, B). 2–4 h post injection, embryos were screened to select for live GFP + embryos with SUM44PE cells only within the PVS (Supplementary Figure S8C). Embryos with deformed yolk sacs or cardiac oedema were excluded, as were those with cells injected into the center of the yolk sac or directly into the circulation (Supplementary Figure S8D). After 24 h, embryos were further screened to exclude very large tumor masses as these embryos had limited survival to end point (Supplementary Figure S8E). Consistent with pathway activation, nuclear STAT3 was detected in IL-6 pre-treated SUM44PE cells in the PVS of embryos at two days post injection (2dpi/4dpf) (Supplementary Figure S8F).

**Fig. 7:**
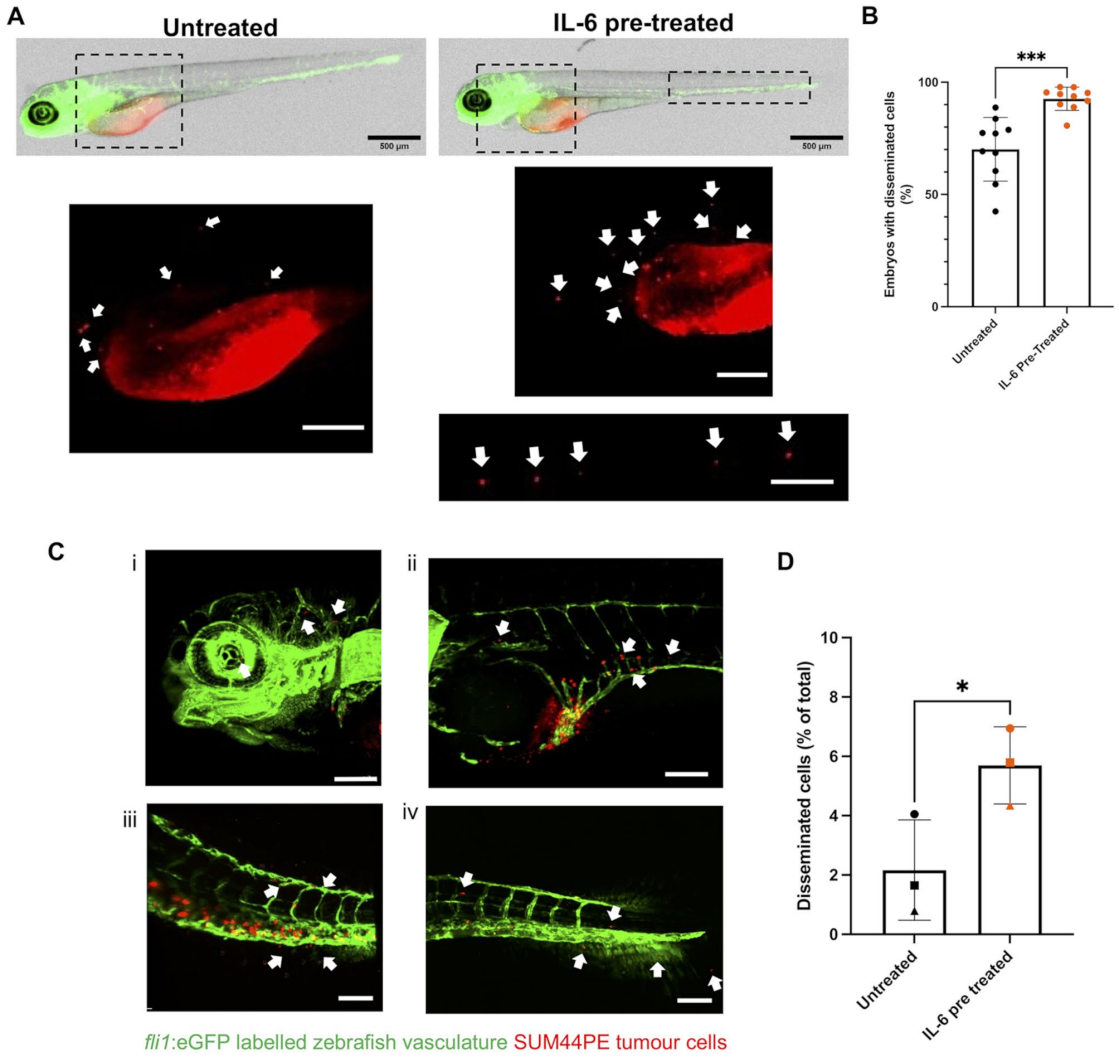
IL-6 promotes increased dissemination of SUM44PE cells in zebrafish embryos. (**A**) Representative images of 2dpi/4dpf Casper *Tg(fli1*:eGFP) Zebrafish embryos injected with untreated (left) or IL-6 pre-treated (right) SUM44PE cells, images on mesoscope at 3.2x, scale bar shows 500 μm with disseminated cells indicated by arrows. Dashed boxes represent zoomed in regions shown below, scale bars show 200 μm. (**B**) Quantification of the percentage of embryos with or without disseminated cells, *n* = 9 independent biological replicates. (**C**) Representative 3D projections of fixed 2dpi/4dpf embryos showing disseminated cells in the (i) head and heart, (ii) the SIV, (iii) CHT and (iv) the tail fin. Arrows indicate cells that have extravasated from the vasculature, z-stacks with 3 μm steps on Andor Dragonfly confocal microscope, scale bars show 100 μm. (**D**) Quantification of the percentage of all SUM44PE cells that are disseminated in the embryos (outside the primary site and extravasated from the vasculature). Quantified using Imaris 10.0.1. *n* = 3 biological replicates, with 3–4 embryos in each group per replicate, points represent the mean of each replicate. (**C**) and (**E**) two-way paired t-tests in GraphPad Prism, * *p* < 0.05, *** *p* < 0.001. *fli1*:eGFP labelled vasculature in green, DiI-dyed SUM44PE cells in red

At 2dpi/4dpf, the percentage of embryos with cells that had disseminated out of the PVS was calculated. There were significantly more embryos with disseminated cells following pre-treatment of the cells with IL-6 (Fig. 7A, B). The experiment was repeated with Luc-ZsGreen expressing SUM44PE cells (Supplementary Fig. S9) and confirmed the findings with DiI-labeled SUM44PE cells (52% and 75% embryos with disseminated cells for untreated and IL-6 pre-treated respectively).

Embryos were then fixed and imaged to quantify the number of disseminated cells. Disseminated SUM44PE cells were found in the heart region, within the subintestinal vein, throughout the head region, the caudal hematopoietic tissue and in the tail fin (Fig. 7C). Significantly more cells that had extravasated from the vasculature at distant sites were found in embryos injected with IL-6 pre-treated SUM44PE cells (Fig. 7D), demonstrating that IL-6 is able to promote increased dissemination of ILC cells in the zebrafish embryo.

## Discussion

ILC tumors have a high stromal content, however, the consequences of this for ILC growth and metastatic capabilities are not known. Here we have identified IL-6 as a CAF-derived factor that mediates crosstalk between the tumor stroma and tumor cells in ILC, leading to activation of the JAK/STAT3 signaling pathway, and increased migration, invasion and dissemination of ILC cells in zebrafish embryos. Activation of the IL-6/JAK/STAT3 pathway has been reported in many tumor types including breast, and additionally elevated serum levels of IL-6 in breast cancer patients have been linked to poor prognosis [39, 40]. However, there are no specific reports of the IL-6/JAK/STAT3 pathway in ILC: our data support paracrine activation of the IL-6/JAK/STAT3 pathway in ILC.

IL-6 can be produced by numerous stromal cells including CAFs, in addition to tumor cells themselves, and both paracrine and autocrine activation of the IL-6/JAK/STAT3 pathway in tumors has been reported. We show here that CAFs isolated from human and mouse ILC express and secrete IL-6, while human and mouse ILC cell lines do not express IL-6. This may reflect the down-regulation of IL-6 by estrogen [41] and is consistent with early reports of IL-6 mRNA in basal-like breast carcinoma tissues, but not in ductal breast carcinoma [42]. Our analysis of human ILC samples further support a paracrine IL-6 signaling pathway in ILC where there is a strong positive correlation between *IL6* and the stromal content of tumors, and a correlation between stromal *IL6* and nuclear STAT3 within the tumor compartment and limited *IL6* expression in the epithelial compartments of both mILC and human ILC tumors in scRNA-Seq datasets. The scRNAseq analysis also demonstrates high *IL6* expression by endothelial cells. Higher microvessel density has been reported in ILC than NST [12], and *PECAM1* (CD31) expression is significantly higher in ILC than NST in the TCGA (data not shown). Whilst we did not explore the role of endothelial cells in this manuscript, endothelial cells may play a role in the IL6/STAT3 pathway in ILC and should be investigated in the future.

Although IL-6/JAK/STAT3 signaling has been reported in ER + NST samples, here we demonstrate that the IL-6/JAK/STAT3 pathway is enriched in ILC compared to NST when analyzing clinical samples both at the gene expression and protein level. This most likely reflects both an increase in stromal content in ILC providing increased expression of *IL6*, and also a specific increase in *IL6* within the stroma. In addition, we identified IL-6 dependent gene signatures that are enriched in ER + ILC compared to ER + NST. This identified genes that are known to be regulated by STAT3 including those previously reported in an IL-6 dependent gene signature in ER + breast cancer lines (*CEBPD*, *IFITM2*, *IFITM3*, *S100A9*, *TMC5*) [43], and some genes that are in the Hallmark IL6-JAK-STAT3 gene set (*HMOX1*, *IL4R*, *IL13RA1*, *STAT3*, *SOCS3*). We also identified several genes which have not previously been reported as STAT3 targets or to be regulated by IL-6 (*LRP2*, *LRRC4C*, *PPP1R1B*, *SLC28A3*, *MUCL1*) suggesting that different transcriptional control could potentially drive ILC-specific gene expression. The relevance of these genes to ILC is not known, but together with the differential association of IL6-JAK-STAT3 genes and our CAF-IL6 genes with survival in ER + ILC compared to ER + NST, this supports an important role for paracrine IL-6 signaling in ER + ILC. Furthermore, it suggests that the transcriptional networks that drive the pro-tumorigenic roles of the IL-6/STAT3 pathway are different in ER + ILC and ER + NST and may be clinically relevant. In ER + NST, STAT3 co-opts shared enhancers to drive a distinct gene program independent of ER and its pioneer factor FOXA1, and this drives metastasis which is uncoupled from ER signaling [29]. Although some of the genes identified were the same as those in the IL-6 gene signature in ILC, it will be important to establish whether a similar mechanism of STAT3-dependent regulation of gene expression is present in ER + ILC.

Recently, a distinct chromatin state in ILC was identified that results in a unique FOXA1-ER axis in ILC that promotes transcription of genes associated with tumor progression and poor outcomes [34]. We found that IL-6 suppresses expression of this gene signature in ILC, implicating IL-6 in the resistance to endocrine therapy in ILC as has been reported previously for ER + ductal tumors [44, 45]. Additionally, analysis of long-term estrogen-deprived human ILC cell lines [46], showed upregulation of STAT3 and genes from the IL6GS, again supporting a role for this pathway in driving resistance to endocrine therapies in ILC.

Consistent with our reports of IL-6 driven migration and dissemination of ILC, a number of the IL6GS genes have been shown to drive migration and metastasis. *AKR1B10* behaves as a metastasis enhancer in breast cancer via its ability to promote fatty acid oxidation, although high expression was only associated with poor distant metastasis free survival in ER- and HER2 + breast cancer, not in ER + breast cancer [47]. In contrast *BCL3* drives metastasis in ERBB2-driven mammary tumors [48], and its expression has been shown to correlate with poor survival in ER + breast cancer, including ILC [49]. Mucins or mucin-like glycoproteins *MUC5B* and *MUCL1* are often deregulated in cancer and are reported to have higher expression in breast cancer than normal tissue [50]. MUC5B and MUCL1 (also known as Small Breast Epithelial Mucin/SBEM) have both been shown to drive invasion in vitro [51–53] and are associated with poor survival and metastasis in breast cancer patients [54–56], although there are no reports on effects in ILC. STAT3 induced expression of the secreted glycoprotein LRG1 promotes invasion and metastasis in colorectal cancer models [57], and LRG1 also induces metastasis of melanoma cells via STAT3 activation [58]. STAT3 is known to suppress apoptosis [39]. However, changes to both pro-and anti-apoptotic genes were seen following IL-6 treatment and as such the impact on the pro-tumorigenic role of IL-6 in ILC is not clear.

ILC is a proportionally under-studied disease, and the lack of models is an issue for research [59]. Although the use of zebrafish has many limitations, patient samples from multiple cancer types implanted into zebrafish embryos have been shown to recapitulate many features and drug responses of both the primary patient tumor and mouse xenografts [60]. The ability to follow dissemination of ILC cells within 2–4 days in zebrafish embryos, when it can take up to a year to establish intraductal ILC xenografts in mice [61], is an exciting advance. Pilot experiments with the MM134 cell line and HCI-013 organoids showed that they were also both viable and able to disseminate, demonstrating the utility of this assay for the study of ILC cell intrinsic mechanisms of metastasis.

A number of approaches are being developed to target the IL-6/JAK/STAT3 pathway some of which have been approved for use in inflammatory diseases such as rheumatoid arthritis [62]. Due to its well-known role in controlling inflammation, it will be important to assess the impact of CAF-derived IL-6 and subsequent downstream signaling on the ILC immune microenvironment. Clinical trials are being undertaken in several tumor types including breast, although as yet they have not been approved for use [21, 39]. Although blocking IL-6 was sufficient to inhibit the CAF-dependent STAT3 activation in our model systems, the CAFs secrete other factors that are known to activate STAT3 (e.g. osteopontin, GM-CSF), and use of a JAK inhibitor such as baricitinib may offer more benefit in the clinical setting. Here we show that the pathway is activated and enriched in ILC, and that it provides multiple pro-tumorigenic signals, suggesting that it may be a potential therapeutic target in ILC. It will be important to understand which IL-6/STAT3 target genes are driving the pro-tumorigenic phenotype in ILC and whether they can be suppressed by drugs that target the IL-6/JAK/STAT3 pathway.

## Materials and methods

### Cells and patient-derived models

SUM44PE (BioIVT) and MDA-MB-134VI (ATCC) were cultured following the suppliers’ instructions. Primary patient-derived CAFs were characterized and grown as previously described [17]. HCI-013 and HCI-018 ER + ILC PDOs were obtained from Professor Alana Welm [22] and the LA-PDxO from Professor Richard Iggo which was established from the LA-PDX1 [33]. Tumor cells (13-MCB-17 and 10-SJK-221) and CAFs (8002 and 9188) isolated from WEPtn mILC tumors were a kind gift from Dr Julia Houthuijzen. Human cell lines were authenticated via short tandem repeat DNA profiling (Culture Collections, UK) and confirmed to be mycoplasma-negative by qPCR performed at the MRC Human Genetics Unit (University of Edinburgh). Details of media used and additional experimental conditions can be found in Supplementary Methods.

### RNA-Seq

RNA was extracted using the Qiagen RNeasy kit with DNAseI digestion. Library preparation was carried out at the Wellcome Trust Clinical Research Facility (University of Edinburgh) using the Lexogen QuantSeq 3`-mRNA-Seq Library Prep kit (FWD) (Lexogen Inc, #015). Single-read sequencing was performed on the NextSeq 550 platform (Illumina Inc, #SY-415-1002) using the NextSeq 500/550 Mid-Output v2.5 (150 cycle) Kit (#20024904). The Illumina Bluebee platform and Lexogen data analysis pipelines were used for alignment and counts for the CAF CM and IL-6 RNA-Seq experiments respectively. Differential gene expression analysis was carried out using the EdgeR package (v3.40.0), with samples normalized by trimmed mean of M-values in R 4.2.3. Raw files and gene counts are available at GSE276108. Differential gene expression analysis results are available in Supplementary File S2. Details of the CAF IL6GS and consensus IL6GS are available in Supplementary File S1.

### Zebrafish xenografts

The zebrafish embryo xenograft assay was carried out following the protocol developed by the Fior group [38, 63]. Casper and Casper *Tg(fli1*:eGFP) zebrafish maintenance was carried out under project license PP7283023 and UK Home Office regulations, UK Animals (Scientific Procedures) Act 1986, amended in 2013, and European Directive 2010/63/EU. All experiments were approved by the University of Edinburgh Animal Welfare and Ethical Review Body. Adult Casper and Casper *Tg(fli1*:eGFP) fish were maintained at 28.5 °C in 14/10 hour light/dark cycles. Prior to injection, embryos were maintained at 28.5 °C and after injection, at 32 °C in E3 media until harvested.

### Clinical samples

Details of the tissue microarray generated in Glasgow from a retrospective cohort of 538 ER + breast cancer tumors has been described previously [27]. An additional cohort of 246 human primary operable ILC tumors was collected under the approval of the research ethics committee of the Queen Elizabeth University Hospital Glasgow (REC reference: 22/WS/0020, IRAS project ID: 306447), and a cohort of ER + ILC and NST cases were retrieved from Sichuan Provincial People’s hospital (Chengdu, China) after approval by the hospital’s ethics committee. Further details in Supplementary Methods.

### Publicly available datasets

All analysis was restricted to ER + ILC and ER + NST samples. TCGA Firehose RNA-Seq RSEM and clinical data was accessed through cbioportal in June 2022. TCGA Firehose RPPA level 4 median normalized and batch corrected data was downloaded from https://tcpaportal.org/tcpa/download.html in April 2020. ESTIMATE stromal scores were accessed in October 2021 from https://bioinformatics.mdanderson.org/estimate/disease.html Laser-capture microdissection data for ILC and NST tumors was obtained from GSE148398 and GSE68744 [17, 26]. scRNA-Seq data of human ILC tumors [23] were accessed through, analyzed and graphs produced using the Broad Institute Single Cell Portal [64]. scRNA-Seq data of WEPtn mILC tumors was from [13]. RATHER ILC [6] cohort mRNA data was obtained from GSE68057 and clinical data from 10.6084/m9.figshare.1301848. *M*ixed non-classical and non-specified histological subtype samples were excluded from analysis.

### Statistical analysis

Statistical analysis was carried out using GraphPad Prism v10.2.0 or R 4.2.3 using RStudio. Analyses were two-sided with p/p-adjusted/FDR < 0.05 considered significant. GSEA of the Hallmark gene sets was carried out using GSEA 4.2.2 desktop application with gene set permutation, FDR < 0.05 was considered significant [30, 31]. Single sample GSVA was carried out using the GSVA R package (v2.0.6) [65]. ssGSVA scores for the CAF IL6GS and consensus IL6GS in ER + TCGA samples are in Supplementary File S3. Survival analysis of TCGA and RATHER cohorts was carried out using survminer (0.5.0) and survival (3.8-3) packages in R. SPSS version 28 (IBM, New York, USA) was used to analyze clinical samples.

### Supplementary methods

Details of additional methods and analysis can be found in Supplementary Data file.

## Electronic supplementary material

Below is the link to the electronic supplementary material.

Supplementary Material 1

## Data Availability

The RNA-Seq datasets generated during the current study are available in the NCBI GEO database, GSE276108.
