# Supplementary material for: Cancer-associated fibroblast driven paracrine IL-6/STAT3 signaling promotes migration and dissemination in invasive lobular carcinoma
Source: Breast Cancer Res. 2025 Jul 1;27:121. doi: 10.1186/s13058-025-02074-x (PMC12219320; doi:10.1186/s13058-025-02074-x)

Cancer-associated fibroblast driven paracrine IL-6/STAT3 signaling promotes migration and dissemination in invasive lobular carcinoma

Supplementary Data

This file contains:

- Index of Supplementary Files
- Supplementary Methods
- Supplementary Methods References
- Supplementary Table S1
- Supplementary Figures
- Raw Western blot images

INDEX OF SUPPLEMENTARY FILES

Supplementary File S1 – Genes in CAF and consensus IL6GS

Supplementary File S2 – Results of differential gene expression analysis for SUM44PE + CAF CM +/- anti-IL-6 (S2-1), SUM44PE + IL-6 (S2-2), MM134 + IL-6 (S2-3), HCI-013 + IL-6 (S2-4), HCI-018 + IL-6 (S2-5)

Supplementary File S3 – ssGSVA scores for TCGA samples for CAF IL6GS and Consensus IL6GS

Supplementary File S4 – Antibodies used for RPPA (S4-1) and FPPA (S4-2)

SUPPLEMENTARY METHODS

Cell culture

**Cell line**	**Media**
SUM44PE	Complete media<br>Ham's F-12 (Gibco 11-765-054)<br>BSA (1 g/l, ThermoFisher Scientific 15260037)<br>Ethanolamine (5 mM, Sigma-Aldrich E0135)<br>HEPES (10 mM, Sigma-Aldrich H3375)<br>Hydrocortisone (1 µg/ml, Sigma-Aldrich H4001)<br>Insulin (5 µg/ml, Sigma-Aldrich I9278)<br>Sodium Selenite (50 nM, Sigma-Aldrich S7133)<br>Apo-transferrin (5 µg/ml, Sigma-Aldrich T2252)<br>Triiodo thyronine (10 nM, Sigma-Aldrich T5516)<br><br>Growth factor depleted media<br>As above without insulin, hydrocortisone or triiodo thyronine in phenol-red free DMEM/F-12 (ThermoFisher 21041025)<br><br>2X concentrated growth factor depleted media<br>Phenol-red free DMEM/F-12 (ThermoFisher 21041025)<br>BSA (2 g/l)<br>Ethanolamine (10 mM)<br>HEPES (20 mM)<br>Sodium Selenite (100 nM)<br>Apo-transferrin (10 µg/ml)
MDA-MB-134VI	1:1 DMEM (Sigma Aldrich D6429):L15 (ThermoFisher Scientific 11415064) + 10% FBS<br><br>Growth factor depleted media<br>1:1 DMEM phenol red free (ThermoFisher Scientific 21063029): L-15 (ThermoFisher Scientific 21083027)
HCI-013/HCI-018 PDOs	As described in Scherer *et al,* 2023 (10.1016/j.xpro.2023.102402)<br>Grown in phenol red free growth factor reduced Matrigel (VWR, 734-1101)
WEPtn tumor cells (13-MCB-17, 10-SJK-221)	DMEM/F12 (Sigma Aldrich D8437)<br>10% FBS<br>Mouse EGF (5 ng/mL, Sigma Aldrich E9644)<br>Cholera Toxin (5 ng/mL, Sigma Aldrich, C8052)<br>Insulin (5 µg/ml, Sigma-Aldrich I9278)
Human CAFs	DMEM/F-12 + 10% FBS<br>On collagen coated plates – Bovine Collagen I (Biomatrix #5005) diluted in dH2O to 100 µg/ml, with roughly 5 µg/cm^2<br><br>Conditioned media<br>Phenol-red free DMEM/F12 (ThermoFisher Scientific 21041025)
WEPtn CAFs (8002 and 9188)	DMEM high glucose<br>20% FBS<br>Penicillin/streptomycin 1% (ThermoFisher Scientific 15140122)

LA-PDX1	Advanced DMEM/F12<br>Primocin® (100 mg/mL)<br>HEPES (10 mM)<br>Glutamax (2 mM)<br>B27 (1X)<br>Rspo1 (1X)<br>Noggin® (1X)<br>FGF7 (100 ng/mL)<br>FGF10 (100 ng/mL)<br>HGF (100 ng/mL)<br>NRG4 (100 ng/mL)<br>AREG (100 ng/mL)<br>A83-01 (500 nM)<br>SB202190 (10 μM)<br>PGE2 (1 μM)<br>Nicotinamide (10 mM)<br>N-Acetyl-L-Cysteine (1.25 mM)<br>E2 (1 nM)<br>Y-27632 (10 uM)

SUM44PE Luc-ZsGreen cells were made by lentiviral transduction of SUM44PE cells with pHIV-LucZsGreen (Addgene 39196) and sorted for ZsGreen expression using a Cytoflex SRT sorter (Beckman Coulter).

Human cells were cultured in growth factor depleted and phenol-red free media for 24 hours before stimulation with recombinant human IL-6 (10 ng/mL, Biolegend, USA, #570804), CAF conditioned media, anti-human IL-6 neutralizing antibody (1 μg/mL, Biolegend #501125) or baricitinib (125 nM, APExBIO, #A4141). For CAF conditioned media stimulation of SUM44PE and MM134 cells, conditioned media was diluted 1:1 in SUM44PE 2X concentrated growth factor depleted media or MM134 growth factor depleted media, respectively.

WEPtn mILC tumor cells were serum starved for 24 hours before stimulation with recombinant mouse IL-6 (20 ng/mL, Biolegend, #575706) or anti-mouse IL-6 neutralizing antibody (1μg/mL, Biolegend, #504512).

siRNA

C12-200 lipoparticle siLuciferase (siCTRL) and siSTAT3 were a kind gift from Dr Lidia Avalle, University of Torino, Italy. Cells were treated with 1 μg/mL siCTRL/siSTAT3 for 48 hours [1].

Conditioned media

To collect conditioned media (CM), cells were serum starved in phenol-red free media and media collected after 72 hours. CM was collected, centrifuged at 1700 rpm for 10 min at 4°C to remove debris then filtered through 0.22 µm filter. CM was diluted 1:1 with the appropriate growth factor depleted media when used to stimulate cells.

IL-6 ELISA

CM was collected from SUM44PE, MDA-MB-134-VI, primary human CAFs and WEPtn mILC tumor cells and CAFs after 72 hours. Human CAF CM was diluted 1:10 and human tumor, WEPtn tumor and CAF CM was added undiluted to the capture antibody coated plate. The Biolegend ELISA Max Deluxe Set Human IL-6 (#430504) or Mouse IL-6 (#431304) kit was used and was carried out following manufacturers' instructions. CAFs were lysed after collection of the CM, protein content determined and used to normalize IL-6 concentrations.

Immunoblotting

Cells were washed once in ice cold PBS then lysed on ice with RIPA buffer with protease and phosphatase inhibitors for 15 minutes, sonicated using a Bioruptor sonicator with 3 cycles of 30 seconds on and 30 seconds off on high at 4°C, then centrifuged at maximum speed at 4°C for 15 minutes. Samples were prepared at 1 µg/µl and run on a 12-15% SDS-PAGE gel then transferred onto a PVDF membrane. Blots were blocked in 5% bovine serum albumin (BSA) in TBS + 0.1% Tween20 (TBST) then incubated with primary antibodies overnight at 4°C. Blots were washed in TBST, incubated in HRP-conjugated secondary antibodies in TBST + 5% BSA at room temperature for 45 minutes, washed in TBST then developed using Bio-Rad ECL and imaged on Bio-Rad image detection system.

Primary antibodies: STAT3 (1:1000, Cell Signaling Technology (CST), 12640), STAT3 pY705 (1:1000, CST, 9145), GAPDH (1:1000, CST, 5174), cofilin (1:10 000, CST, 5175), ERK1/2 (1:1000, CST, 9102), ERK1/2 pT202/Y204 (1:1000, CST, 9101). Secondary antibodies: anti-rabbit-HRP (1:5000, CST 7074), anti-mouse-HRP (1:5000, CST, 7076).

Immunofluorescence

Cells were seeded onto glass coverslips and after experiments were carried out, cells were washed once with 1X TBS and fixed for 10 min at room temperature with PIPES/formaldehyde

fixation buffer (3.7% formaldehyde, 100 mM PIPES (Sigma Aldrich P6757), 10 mM EGTA (Millipore 324626), 1 mM $MgCl_2$, 0.2% Triton X-100, dH_2O to 10 mL). Formaldehyde was quenched for 10 min in 0.1 M glycine then washed twice in TBS + 0.1% Triton X-100 (TBS-Tr). Samples were incubated for 30 min in block buffer (TBS-Tr + 2% BSA) then incubated overnight at 4°C with primary anti-STAT3 (1:500, CST 12640) or anti-ERα (1:200, CST 13258) diluted in block buffer. Samples were washed three times in TBS-Tr, then incubated with fluorophore-conjugated secondary antibodies (Alexa-Fluor-488 anti-rabbit, 1:500, Invitrogen A11008) and Phalloidin Atto-647N (1:400, Sigma Aldrich 65906) diluted in block buffer for 45 min at RT in the dark. Samples were washed three times in TBS-Tr, once in distilled H2O then mounted onto coverslips using Vectashield with DAPI (Vector Laboratories, #H-1200). Confocal microscopy was carried out using an Olympus FV3000 Confocal Laser Scanning Microscope. Image analysis was carried out in ImageJ - to determine nuclear intensity of STAT3 and ERα, a mask of the nuclei on the DAPI channel was generated and applied to the STAT3/ERα channel. The mean gray value of STAT3 or ERα in the nucleus of each cell in the image and average across the entire image was calculated in at least three fields of view per experiment. The mean gray values were normalized to the mean across all control cells.

RT-qPCR

RNA was extracted using Qiagen RNeasy mini kit with DNase I digestion following manufacturers' instructions. 1 µg of RNA was used to synthesize cDNA using the Superscript First Strand Synthesis System for RT-PCR (Thermo Fisher, #11904018) with random hexamers. qPCR was carried out using SYBR select master mix (Thermo Fisher, # 4472908) with 1 µL cDNA and 100 nM of forward and reverse primers (see below). Three biological replicates and two technical duplicates were analyzed for each condition with expression normalized to *GAPDH* expression and fold change relative to control presented.

Primer sequences were as below:

**Target**	**Forward primer**	**Reverse primer**
STAT3	GGAACAAGCCCCAACCGGA	CTAAAATCAGGGGTCCCAACTGT
S100A8	TGCTAGAGACCGAGTGTCCTC	TGCCACGCCCATCTTTAT
S100A9	TCCCACGAGAAGATGCACGA	TGGCCACTGTGGTCTTAGGG
MUCL1	GCGCCTTGCCTTCTCTTAGG	GCTGTTGTCGGATTCTGGGC
SERPINB5	CCTCCACATCCAGGTCTTTGT	TTGCTAGTTGCAGGGCATCCAT
GAPDH	GGACCTGACCTGCCGTCTAG	TGGTGCTCAGTGTAGCCCAG

CEBPD	GCCATGTACGACGACGAGA	TTGCTGTTGAAGAGGTCGG
BCL3	CCGGAGGCGCTTTACTACC	TAGGGGTGTAGGCAGGTTCAC
LRG1	AACACACGATGGGCTTTCCT	ATCTGGGAAACAGGGAACGG

RNAseq sample generation

For the CAF CM RNA-Seq experiment, SUM44PE cells were grown for 24 hours in phenol red free growth factor depleted media then stimulated for 24 hours with ED26 primary ILC CAF conditioned media +/- 1 µg/mL IL-6 neutralizing antibody (Biolegend, #501125). For the IL-6 only RNA-Seq experiments, SUM44PE cells were grown in growth factor depleted media for 24 hours and MDA-MB-134VI cells and PDOs were grown in phenol-red free media with charcoal stripped FBS for 24 or 72 hours respectively and stimulated with recombinant human IL-6 for 24 hours.

Reverse phase protein arrays

SUM44PE cells were growth factor starved for 24 hours then stimulated with ED38 ILC CAF conditioned media diluted 1:2 in phenol red free growth factor depleted media for 30 min or 24 hours and RPPA carried out as described previously [2] at the HTPU, University of Edinburgh. The background intensity was subtracted from signal intensity of each antibody then normalized to total protein fast green protein stain intensity. Differential expression was determined by two-way ANOVA with Benjamini, Kreiger, Yeiketieli (BKY) multiple comparison correction in GraphPad Prism, FDR<0.05. The list of antibodies used can be found in Supplementary File 4.

Forward phase array

$4x10^5$ CAFs were seeded on collagen coated 10 cm dishes in complete media. After 24 hours, media was changed to 4 mL DMEM/F12 phenol red free for 72 hours. Conditioned media was collected as above and stored at -80°C until use. Forward phase array was carried out as described previously with validated capture-detection antibodies (Supplementary File 4) [3]. Biotin labeled secondary antibodies were detected with fluorescently labeled streptavidin and fluorescence intensity was analyzed using an Innopsys 710 slide scanner and Mapix software. Average signal intensity for each protein was determined and median background intensity was subtracted. Intensity of spots on a media only slide was then subtracted from each sample.

Intensity was normalized to cell pellet concentration determined by BCA and proteins with a signal above 0 in at least 3 samples were considered above threshold.

scRNA-Seq data analysis

Processing of WEPtn scRNA-Seq data was performed using Seurat version 4.3.0.1. Cells with a minimum of 200 genes and genes present in minimal three cells are included in the analysis. Remaining cells with a count number outside 800 and 6000 were filtered out to exclude doublets and damaged cells. Furthermore, cells containing higher than 20% mitochondrial counts were removed. A nearest neighbor graph was computed using 25 PC's, determined using 85% explained variance on the PCA. Clustering was performed with the Louvain algorithm on resolution 0.4. Finally, the data was visualized in a UMAP with the before mentioned 85% explained variance. Human ILC scRNAseq data from Wu *et al* 2021 [4] were accessed through, analyzed and figures generated through Broad Institute Single Cell Portal [5].

EdU proliferation assay

$1x10^5$ SUM44PE cells or $5x10^4$ WEPtn tumor cells were seeded on glass coverslips in 12-well plates. Cells were cultured in growth factor depleted media or serum for 24 hours then stimulated with IL-6 (10 ng/ml human IL-6 or 20 ng/ml mouse IL-6) for 24 hours. For SUM44PE cells, EdU (10 µM, Sigma, #900584) was added to media at the same time as IL-6, for WEPtn, EdU was added to media for the final 2.5 h of IL-6 stimulation, due to differences in speed of replication between the human and mouse cell lines. Cells were then washed 3x PBS, fixed in 4% formaldehyde (Thermo Fisher Scientific, #28908) for 10 min at 37°C then washed 2x PBS. The reaction was stopped with 50 mM NH_4Cl at RT for 10 minutes and washed 2x PBS. Cells were permeabilized for 10 min at RT in PBS + 0.1% Triton X-100 then washed 3x PBS. Cells were labelled using TAMRA-azide EdU labeling solution (CuSO4.5H2O (2 mM Sigma Aldrich C2284), TAMRA-azide (8 µM Jena BioScience CLK-AZ109), L-ascorbic acid (20 mg/mL, Sigma Aldrich A92902), diluted in PBS) for 30 min at room temperature in the dark. Cells are then washed in PBS and placed onto microscope slides with Vectashield with DAPI. Slides were imaged using an Olympus FV3000 Confocal Laser Scanning Microscope at 40x, with 6 random FoVs being analyzed per sample and three samples analysed per condition. The number of DAPI+ and EdU+ nuclei per FoV were counted in ImageJ and the

overall percentage of EdU+ cells was determined by taking the average of the 18 FoVs per condition.

CAF derived matrices

The protocol for generating CAF derived matrices (CDMs) was adapted from the method published by Kaukonen et al. [6]. 96-well Imagelock plates (Sartorius, #BA-04857) were first incubated with 0.2% gelatin (Sigma Aldrich, #G1393) diluted in PBS overnight at 4°C, then washed 3x in PBS and cross-linked using 1% glutaraldehyde (Sigma Aldrich, #354400) diluted in PBS for 30 min at room temperature. Plates were then washed and incubated with 1M glycine (Sigma Aldrich, #G8898), then washed in PBS and incubated in DMEM/F12 + 10% FBS + 1% P/S for 30 min at 37°C. 6500 ED26 ILC CAFs at passage 6 were seeded per well. Once confluent, the media was changed to DMEM/F12 + 10% FBS + 1% P/S with 50 µg/mL ascorbic acid (Sigma Aldrich, #A92902) and media was changed every day for 14 days. Ascorbic acid media was made fresh every day.

To remove CAFs and retain the CDMs laid down, CDMs were denuded using extraction buffer (20 mM NH4OH, 0.5% Triton X100 in PBS) at 37°C for 3-5 min, until no intact cells could be seen. Half the extraction buffer was gently removed and replaced with PBS twice, then remaining DNA was degraded using 10 U/mL DNAse1 (Qiagen, #79256) at 37°C for 30 min. Plates were washed twice very gently with PBS then stored in PBS + 1% P/S at 4°C for up to 3 months. Before SUM44PE cells were seeded onto the CDMs, the matrices were washed with PBS twice gently then incubated with heat denatured (heated to 75°C for 3 min then cooled) 2% BSA (Thermo Fisher #15260037) for 1 hour at 37°C. Plates were then washed in PBS and 1000 SUM44PE cells were seeded per well. Cells were allowed to adhere overnight then stimulated as indicated in experiments and brightfield images were taken at 10X using the Incucyte S3 system. Aspect ratio of SUM44PE cells adhered to CDMs was measured in ImageJ, with an aspect ratio>1.7 considered to be 'mesenchymal-like'.

Collagen haptotaxis assay

SUM44PE cells were cultured in complete media +/- IL-6 (10 ng/ml) for 1 week. The underside of 8 µm pore 24-well plate Transwell inserts were coated with 40 µL rat tail Collagen I (Corning, 354236) diluted to 500 µg/ml in complete SUM44PE media and incubated at 37°C

for 1 hour to form gel. Transwell inserts were then inverted and 3x10^5 untreated or IL-6 pre-treated SUM44PE cells were seeded into the top chamber of the collagen coated Transwell, with 300 µL SUM44PE media in the top chamber and 500 µL SUM44PE media in the bottom chamber. After 72 hours, remaining cells in the top chamber were removed using a sterile cotton swab and the cells that had invaded into the collagen gel were fixed in 70% ethanol then mounted onto coverslips with nuclei labeled using Vectashield + DAPI (Vector Laboratories, #H-1200). 3-4 random fields of view were imaged per Transwell using an Olympus FV3000 microscope at 40X and number of nuclei were quantified in ImageJ.

Migration assay

Cells were seeded onto collagen coated (Advanced Biomatrix #5005) ImageLock 96-well plates (Sartorius, #BA-04857) at 1000 cells/well. After 24 hours, the appropriate wells were stimulated with 10 ng/mL IL-6. Cells were imaged every hour for 72 hours on an Incucyte S3, 10X magnification. Time lapse videos from 24 to 72 hours after IL-6 stimulation were analyzed in ImageJ. Images were filtered using the variance filter then analyzed in Trackmate 7.0 [7]. LoG detector was used with particle size at 50 µm, with median filter and subpixel localization. Spots were filtered based on automated quality. The LaP tracker was used with 100 µm max frame-to-frame linkage, 20 µm gap closing and max 2 frame gaps. Tracks were filtered based on automated mean quality.

Wound healing assay

To assess the effect of IL-6 treatment on 13-MCB-17 cell migration *in vitro* in 2D, 13-MCB-17 cells were serum starved and then pre-treated with 20 ng/ml of mouse recombinant IL-6 for 24 hours prior to seeding into 96-well image lock plates. Once the cells formed a monolayer, commonly used wound-scratch assay was employed, where a gap ("wound") was introduced into the middle of the monolayer to induce directional movement of cells from wound edges. After introducing the wound, cells were kept in culture media containing 20 ng/ml recombinant mIL-6. The IncuCyte live-cell imaging system was used to capture images every hour until the wound closed.

Organotypic assay

1x10^6 WEPtn CAFs were suspended in 2mg/ml rat tail collagen 1 extracted from fresh 12-14 adolescent rat tails following the protocol of Timpson *et al*, 2011 [8]. 2.5ml of CAF-collagen mixture was transferred onto 12x35mm plastic dishes and allowed to set for 10 min at 37°C in incubator. 1 mL of WEPtn CAF growth media was added and CAF-collagen matrix was detached from the side of the dish using a pipette. Matrices were allowed to contract for 8-9 days until they fit in 24-well dish. Using blunt forceps, contracted matrices were transferred into 24 well plates and 4x10^4 WEPtn epithelial cells untreated or pre-treated for 24 h with recombinant mIL-6 (20 ng/ml) were plated on top of the matrices. Cells were allowed to grow for 3-5 days until confluency. Stainless steel grids were kept in 6cm dishes and WEPtn epithelial cell growth media was added to a level above the grid. Matrices were kept on the metal grid and media was gently aspirated so the bottom of the matrix is in contact with media but not submerged. After 5 days, the matrix was fixed in 4% formaldehyde overnight and paraffin embedded. The blocks were processed and cut for H&E staining or stained with anti-pan-cytokeratin (1:500, Abcam ab9377) and analyzed at 40X magnification using a Hamamatsu NanoZoomer and viewed on NDP Viewer (Hamamatsu, Hertfordshire, UK).

Clinical cohorts

Glasgow cohort of 246 human primary operable ILC: The primary tumor was available as formalin fixed paraffin-embedded (FFPE) blocks for evaluation. A database including clinicopathological information, adjuvant treatments, recurrence, and survival data was available from Glasgow safe haven (safe haven number: GSH/21/ON/008). ER, PR and HER2 status had been carried out retrospectively in diagnostic labs to ensure standardization of techniques. Nottingham grading system was applied to grade tumors. The tumor microarray (TMA) for this cohort was constructed by the Glasgow Tissue Research Facility (University of Glasgow). Briefly, FFPE was obtained from pathology services, and full sections were cut, hematoxylin and eosin (H&E) stained and marked up by pathologist Prof Elizabeth Mallon to pick tumor rich regions. Three 0.6 mm cores were selected from each block and embedded into three separate paraffin blocks. Cores from other tissue types were also embedded on the same block as a positive control during staining. TMA maps were created in such a way that each core could be uniquely identified by a TMA-ID, anonymously connecting it to the information stored in the cohort database.

Chengdu cohort of ER+ treatment naïve NST (73) and ILC (99): Tumors were matched for size and grade and FFPE tumor blocks collected. H&E staining and immunohistochemistry (IHC) staining of E-cadherin together with original pathology reports were then reviewed by two senior pathologists to reconfirm pathological diagnoses.

IHC and RNAscope

STAT3 staining was performed on sections from FFPE tumor blocks. Slides were incubated at 60°C for 1 h following deparaffinization by xylene and rehydration by a series of descending concentrations of alcohol. After heat induced antigen retrieval, slides were blocked in 3% hydrogen peroxide and then 10% normal horse serum. Slides were incubated at 4 °C overnight with STAT3 antibody (D3Z2G Rabbit mAb #12640 1:100, CST) and after incubation with secondary antibody (EnVision+HRP #K4001, Dako) and DAB development, slides were counterstained with hematoxylin. Slides were scanned by a digital pathology slide scanner (Leica Aperio CS2). Protein expression levels were quantified by weighted histoscore as described previously [9]. In situ-hybridization detection for *IL6* and *PPIB* (housekeeping gene) (Advanced Cell Diagnostics, Hayward, CA, USA) mRNA was performed using RNAscope 2.5 LS (Brown) detection kit (Advanced Cell Diagnostics, Hayward, CA, USA) on freshly cut TMA slides. HALO™ ImageAnalysis Software (PerkinElmer, Waltham, MA, USA) was used to quantify the number of probe copies per μm^2 for each tissue core in tumor and stroma separately. For each patient, the means of the three cores were calculated for the *IL6* and *PPIB* probes. The quantity of *IL6* was normalized by dividing by the number of *PPIB* copies in each location to give a ratio.

Survival analysis

For clinical cohort survival analysis: Kaplan Meier Log rank survival analysis was carried out to assess the association between protein expression and disease-free survival (DFS). Cut-points were determined using the Survminer package in R. Cox regression survival analysis was performed between each clinical factor of interest and prognostic factor, and measured as hazard ratio (HR) with 95% confidence interval (CI). Multivariate analysis with the backward conditional model was performed for the variables that were statistically significant on

univariate analysis ($p<0.05$). All analysis carried out using SPSS version 28 (IBM, New York, USA).

For TCGA and RATHER cohort survival analysis: Cut-points were determined using the Survminer package in R and association with overall, progression-free, breast cancer specific and recurrence free survival was carried out using the survival package in R.

Zebrafish embryo xenograft assay

SUM44PE cells were cultured for 1 week +/- IL-6 (10 ng/ml) in complete media. On the day of injection, cells were detached and then $2x10^6$ cells were resuspended in 1.5 mL PBS with 3 µL Vybrant CM-DiI dye (Thermo Scientific, #V22888) and incubated at 37°C for 15 mins to label cells. Cells were resuspended in PBS at $5x10^5$ cells/µL. Roughly 300-400 DiI-labeled cells were injected into the perivitelline space of 2-day post-fertilization (2 dpf) Casper *Tg(fli1*:eGFP) zebrafish embryos anaesthetized in 1X Tricaine (MS-222 0.1 g/L), or for SUM44PE Luc-ZsGreen cells, unlabeled cells were injected into 2dpf Casper embryos.

2-4 hours post injection, embryos were screened to exclude dead embryos, embryos with developmental defects, no injected tumor cells or tumor cells injected directly into the circulation. 1 day post injection (1 dpi/3 dpf), embryos were further screened to exclude dead embryos and those with extremely large tumors or yolk defects. Screening of anaesthetized embryos at 2-4 hours post injection and 1 dpi was carried out at 3.2X magnification using a M205 FCA mesoscope with cMOS camera and 1x objective.

At 2 dpi/4 dpf, embryos were anaesthetized in 1X Tricaine and each individual embryo was assessed at 3.2X for the presence of disseminated tumor cells on the Leica M205 FCA mesoscope with cMOS camera and 1x objective. Embryos were considered to have disseminated tumor cells (MET group) if any tumor cells could be detected outside of the yolk sac using the mesoscope at 3.2x. Embryos were then euthanized in 10X Tricaine, then fixed at in PIPES/paraformaldehyde (100 mM PIPES, 1 mM $MgSO_4$, 2 mM EGTA, 1.5% paraformaldehyde) fixation buffer for 4 hours at room temperature or overnight at 4°C.

Zebrafish embryo image analysis

Confocal microscopy was carried out on fixed embryos, either with no additional staining or with acetone permeabilization and Hoechst staining, using an Andor Dragonfly spinning disc confocal microscope. A montage of Z-stacks with 3 μm steps were taken at 20X of the entire embryos. 3D projections of the entire embryos were analyzed in Imaris 9.7.2. A surface was rendered manually of the entire yolk sac region and surfaces were generated automatically for the GFP-labelled vasculature. For the vasculature surface, smooth surface detail was set at 1.5 μm with background subtraction and local contrast at 3 μm. A threshold of 4.5 μm^2 was used and regions were only kept with voxels greater than 1000. SUM44PE cells were detected automatically as 'spots' by the Imaris software. XY-dimension of spots was set as 3 μm and Z-dimension as 6 μm with background subtraction. Spots were filtered for a mean intensity greater than 240. The total number of spots throughout the entire embryo was recorded and spots outside of both the yolk and vasculature surfaces were designated as disseminated cells. The percentage of disseminated SUM44PE cells relative to total SUM44PE cells was determined for each embryo.

Reference list

1. Avalle, L., et al., *Liver-Specific siRNA-Mediated Stat3 or C3 Knockdown Improves the Outcome of Experimental Autoimmune Myocarditis.* Mol Ther Methods Clin Dev, 2020. **18**: p. 62-72.
2. Macleod, K.G., B. Serrels, and N.O. Carragher, *Reverse Phase Protein Arrays and Drug Discovery.* Methods Mol Biol, 2017. **1647**: p. 153-169.
3. MacLeod, K., et al., *Predicting Response to Radiotherapy in Cancer-Induced Bone Pain: Cytokines as a Potential Biomarker?* Clin Oncol (R Coll Radiol), 2020. **32**(10): p. e203-e208.
4. Wu, S.Z., et al., *A single-cell and spatially resolved atlas of human breast cancers.* Nat Genet, 2021. **53**(9): p. 1334-1347.
5. Tarhan, L., et al., *Single Cell Portal: an interactive home for single-cell genomics data.* bioRxiv, 2023: p. 2023.07.13.548886.
6. Kaukonen, R., et al., *Cell-derived matrices for studying cell proliferation and directional migration in a complex 3D microenvironment.* Nat Protoc, 2017. **12**(11): p. 2376-2390.
7. Ershov, D., et al., *TrackMate 7: integrating state-of-the-art segmentation algorithms into tracking pipelines.* Nat Methods, 2022. **19**(7): p. 829-832.
8. Timpson, P., et al., *Organotypic collagen I assay: a malleable platform to assess cell behaviour in a 3-dimensional context.* J Vis Exp, 2011(56): p. e3089.
9. Morrow, E., et al., *High expression of STAT3 within the tumour-associated stroma predicts poor outcome in breast cancer patients.* Cancer Med, 2023. **12**(12): p. 13225-13240.

SUPPLEMENTARY TABLE S1

Characteristics	Univariate analysis (Cytoplasmic STAT3)		Multivariate analysis (Cytoplasmic STAT3)	
	HR (95% CI)	P value	HR (95% CI)	P value
**Age**				
≤50 years	1		1	
>50 years	3.904(1.966-7.75)	<0.001	4.178(1.884-9.26)	0.001
**Grade**				
I	1		-	
II	0.712(0.0921-5.54)	0.746		0.921
III	0.678(0.349-1.315)	0.256		0.090
**Tumour size**				
≤20mm	1			
21-49mm	1.194(0.766-1.861)			0.111
≥50mm		0.434	-	
**Nodal status**				
Negative	1		-	
Positive	1.724(1.007-2.95)	0.047		0.609
**Lymphovascular Invasion**				
No			-	
Yes	1			
	0.72(0.260-1.99)	0.527		0.847
**Recurrence**				
No	1		1	
Yes	4.292(3.16-5.816)	0.001	3.14(1.98-4.86)	<0.001
**Cytoplasmic STAT3**				
Low	1		1	
High	1.986(1.093-3.607)	0.024	1.940(1.050-3.58)	0.034

Supplementary Table S1. Survival analysis for cytoplasmic STAT3 and other prognostic factors. Univariate and multivariate Cox regression analysis of the relationship between cytoplasmic STAT3, other known prognostic clinicopathological factors and disease-free survival. Factors with a significance of $p<0.05$ on univariate analysis were entered into the multivariate analysis, performed in SPSS.

Supplementary Figure S1

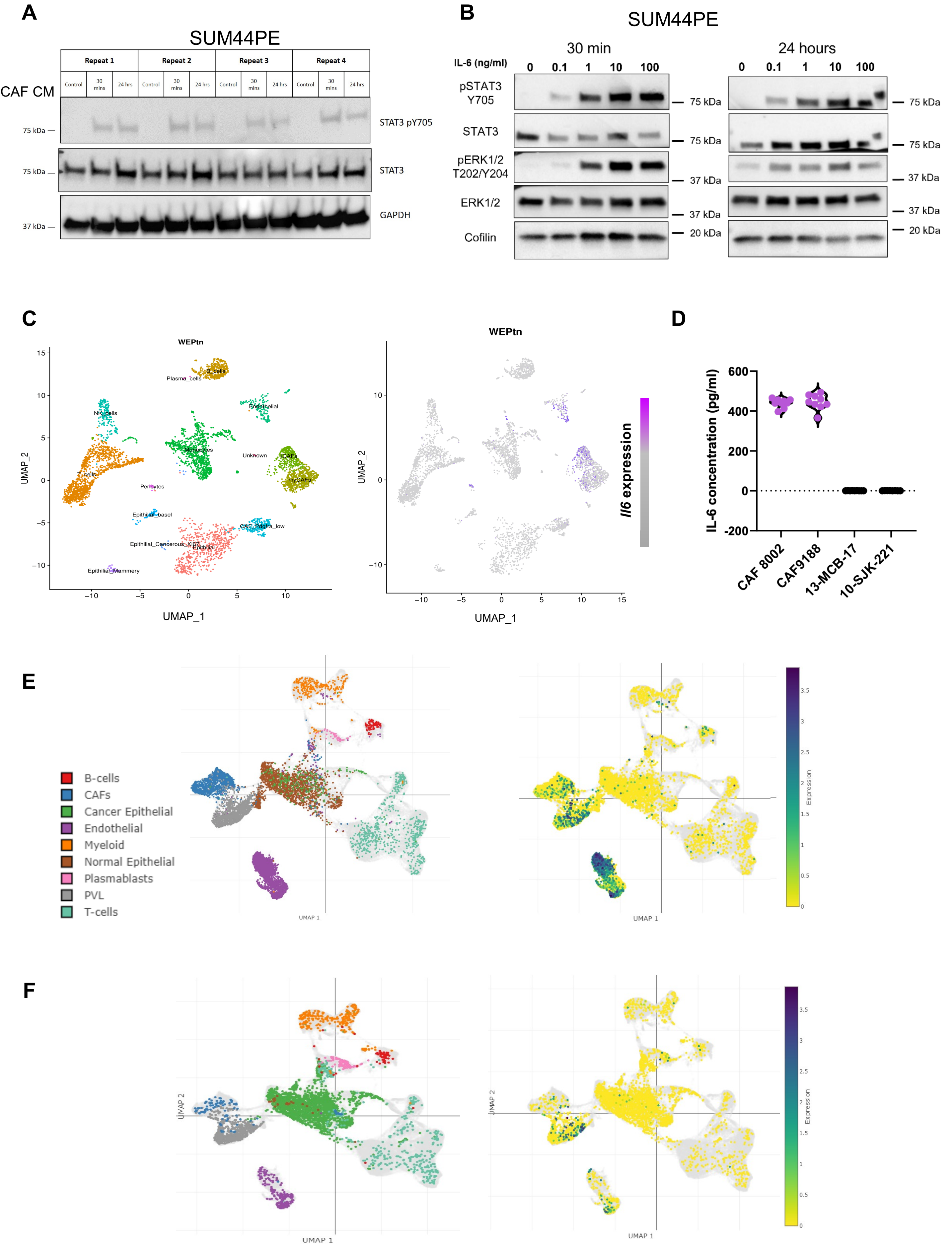

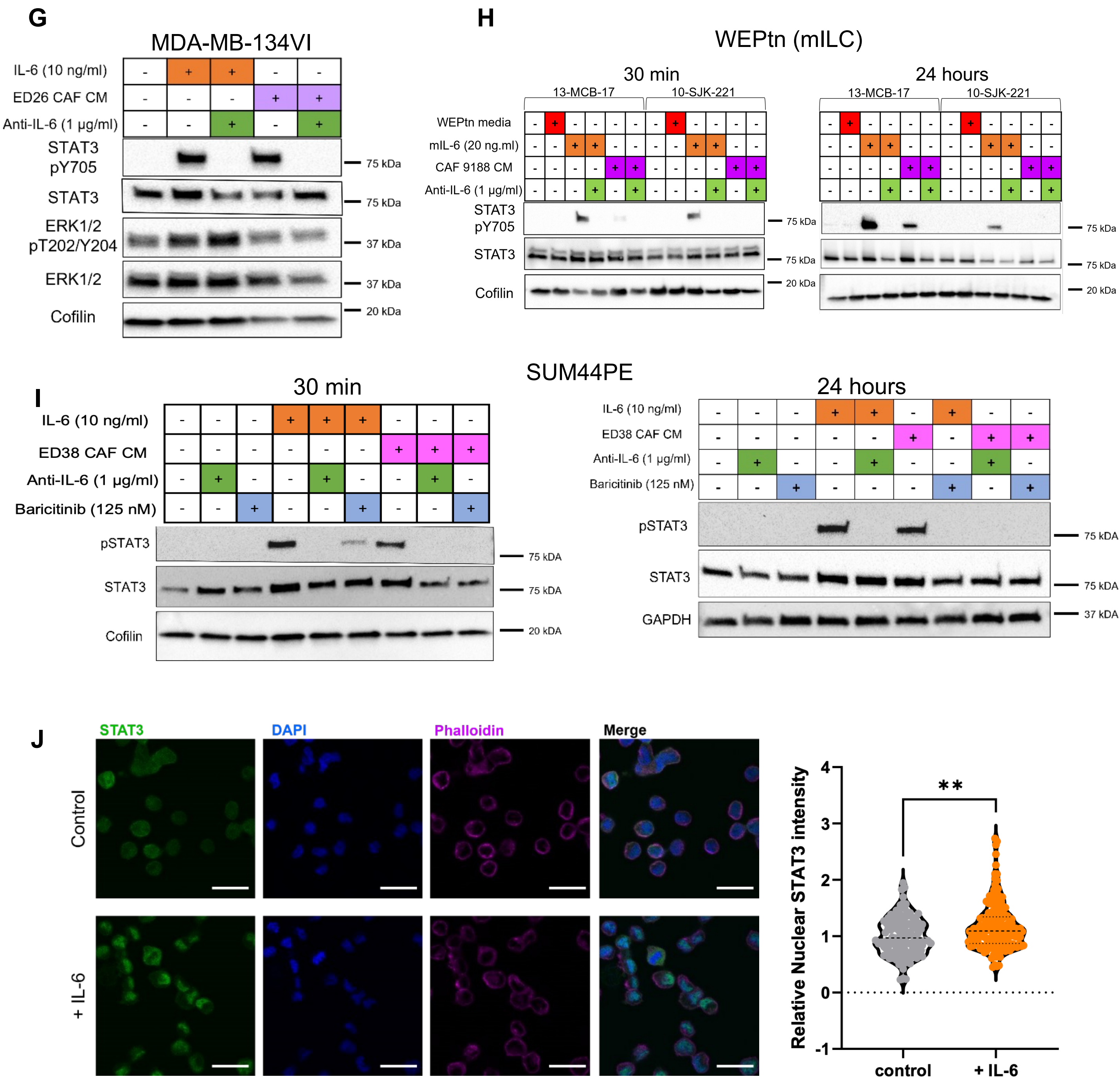
G
MDA-MB-134VI
IL-6 (10 ng/ml)
ED26 CAF CM
Anti-IL-6 (1 µg/ml)
STAT3 pY705
STAT3
ERK1/2 pT202/Y204
ERK1/2
Cofilin
75 kDa
37 kDa
20 kDa
H
WEPtn (mILC)
30 min
24 hours
13-MCB-17
10-SJK-221
WEPtn media
mIL-6 (20 ng.ml)
CAF 9188 CM
Anti-IL-6 (1 µg/ml)
STAT3 pY705
STAT3
Cofilin
SUM44PE
I
30 min
24 hours
IL-6 (10 ng/ml)
ED38 CAF CM
Anti-IL-6 (1 µg/ml)
Baricitinib (125 nM)
pSTAT3
STAT3
Cofilin
GAPDH
75 kDA
37 kDA
20 kDA
J
STAT3
DAPI
Phalloidin
Merge
Control
+ IL-6
Relative Nuclear STAT3 intensity
**
control
+ IL-6

Supplementary Figure S1 Activation of STAT3 by IL-6. A Validation of STAT3 activation in SUM44PE cells by conditioned media (CM) following western blot of cell lysates used for the RPPA. **B** Western blots of SUM44PE cells stimulated with increasing concentrations of recombinant IL-6 for 30 min (left) or 24 hours (right). **C** scRNAseq analysis of WEPtn ILC mouse model tumors showing UMAP clustering (left) and *Il6* expression (right). **D** Concentration of IL-6 in conditioned media (CM) collected from primary mouse ILC CAFs (9188 and 8002) and ILC tumour cells (13-MCB-17 and 10-SJK-221) determined by ELISA. n=1 biological replicate, with technical replicates shown. **E** scRNAseq analysis of human ILC tumours showing (left) UMAP clustering and (right) *IL6* expression in a normal-like ILC tumor (patient 4471) and **F** Luminal B ILC tumor (patient 4535). Data from Wu *et al* 2021, data accessed and figures generated through Broad Institute Single Cell Portal. **G** Western blot of MM134 ILC cell line stimulated with IL-6 (10 ng/mL) or ILC CAF-CM +/- anti-IL-6 (1 µg/mL) for 30 minutes. **H** Western blot of 13-MCB-17 and 10-SJK-221 ILC tumour cells stimulated for 30 min and 24 hours with recombinant human IL-6 or CAF-CM from primary ILC CAFs (9188) +/- anti-IL-6 (1 µg/mL). **I** Western blot of SUM44PE cells stimulated with IL-6 (10 ng/ml) or ED38 CAF CM +/- anti-IL-6 (1 µg/ml) or the JAK inhibitor baricitinib (125 nM) for 30 min (left) or 24 hours (right). **J** Left - representative images of SUM44PE cells stimulated with recombinant IL-6 for 30 min and stained for STAT3 (green), DAPI (blue) and Phalloidin (magenta), 40X magnification, scale bar 25 µm. Right - Relative nuclear STAT3 intensity (mean gray value normalised to mean of control mean gray values) was quantified in ImageJ, n=3 biological replicates for each condition with three random FoVs quantified for each repeat. Each spot represents one nuclei, bar shows mean, two- tailed Mann Whitney U test ** p=0.0013 in GraphPad Prism.

Supplementary Figure S2

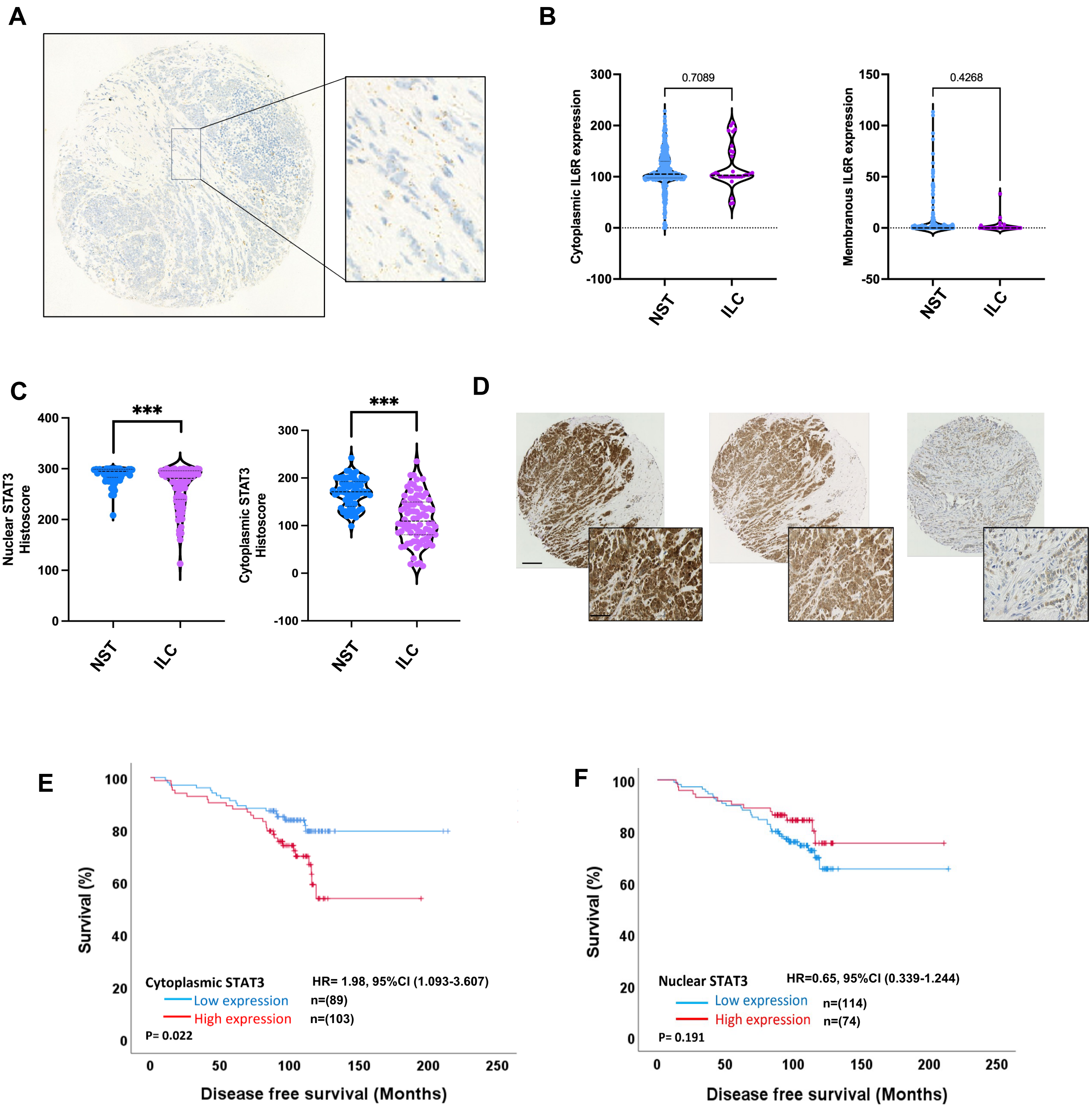

Supplementary Figure S2 IL-6/STAT3 in Glasgow and Chengdu patient cohorts. A Representative *IL6* RNAscope in the Glasgow ILC/NST cohort. **B** Expression of IL6R in the Glasgow ILC/NST cohort, left – cytoplasmic IL6R, right – membranous IL6R (NST=405, ILC n=34), two-tailed Mann-Whitney in SPSS. **C** Expression of STAT3 in the Chengdu cohort in therapy naïve NST (n=72) and ILC (n=82) tumours. Left – nuclear STAT3 expression, right – cytoplasmic STAT3 expression, ** $p<0.001$ for both, two-tailed Mann-Whitney in SPSS. Glasgow ILC Cohort: **D** Representative images of STAT3 staining with – from left to right - predominantly strong cytoplasmic and strong nuclear staining, with predominantly moderate cytoplasmic and moderate nuclear staining, and with predominantly weak cytoplasmic and weak nuclear staining. Scale bars, 200 µm with an inset of 50 µm. Kaplan Meier curves showing the association between disease free survival and **E** cytoplasmic STAT3 ($p=0.022$) and **F** nuclear STAT3 ($p= 0.191$).

Supplementary Figure S3

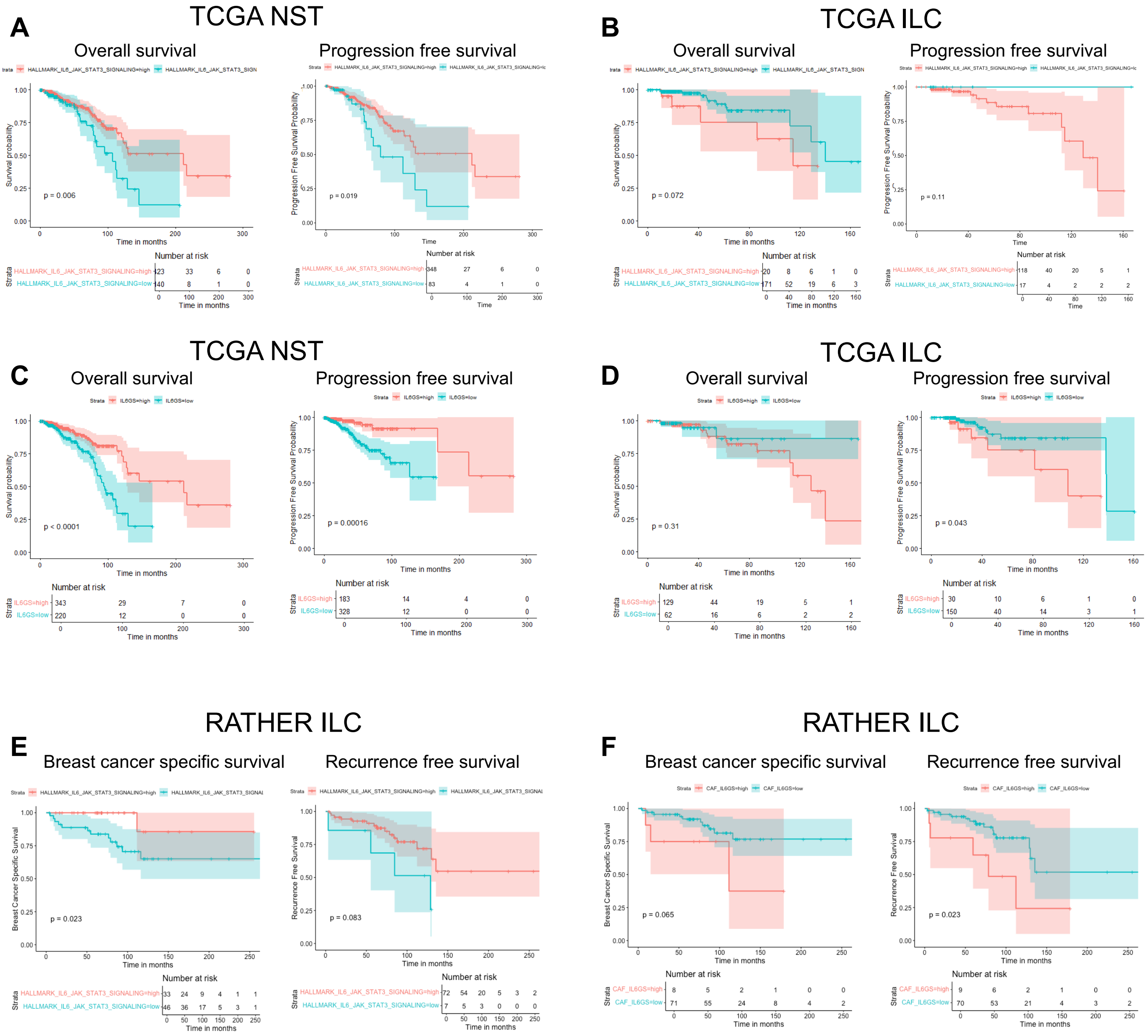

Supplementary Figure S3 Survival analysis of TCGA and RATHER cohorts. Kaplan Meier plots showing association of the **(A, B, E)** Hallmark IL6-JAK-STAT3 ssGSVA score and **(C, D, F)** CAF-IL6GS ssGSVA score with survival in **A** and **C** ER+ NST and **B** and **D** ER+ ILC patients in TCGA and **E** and **F** ER+ ILC patients in the RATHER cohort. **(A-D)** left – overall survival, right - progression free survival, **(E-F)** left – breast cancer specific survival, right – recurrence free survival. Cut points generated by Survminer package in R, log rank p-value displayed as determined by Survival package in R.

Supplementary Figure S4

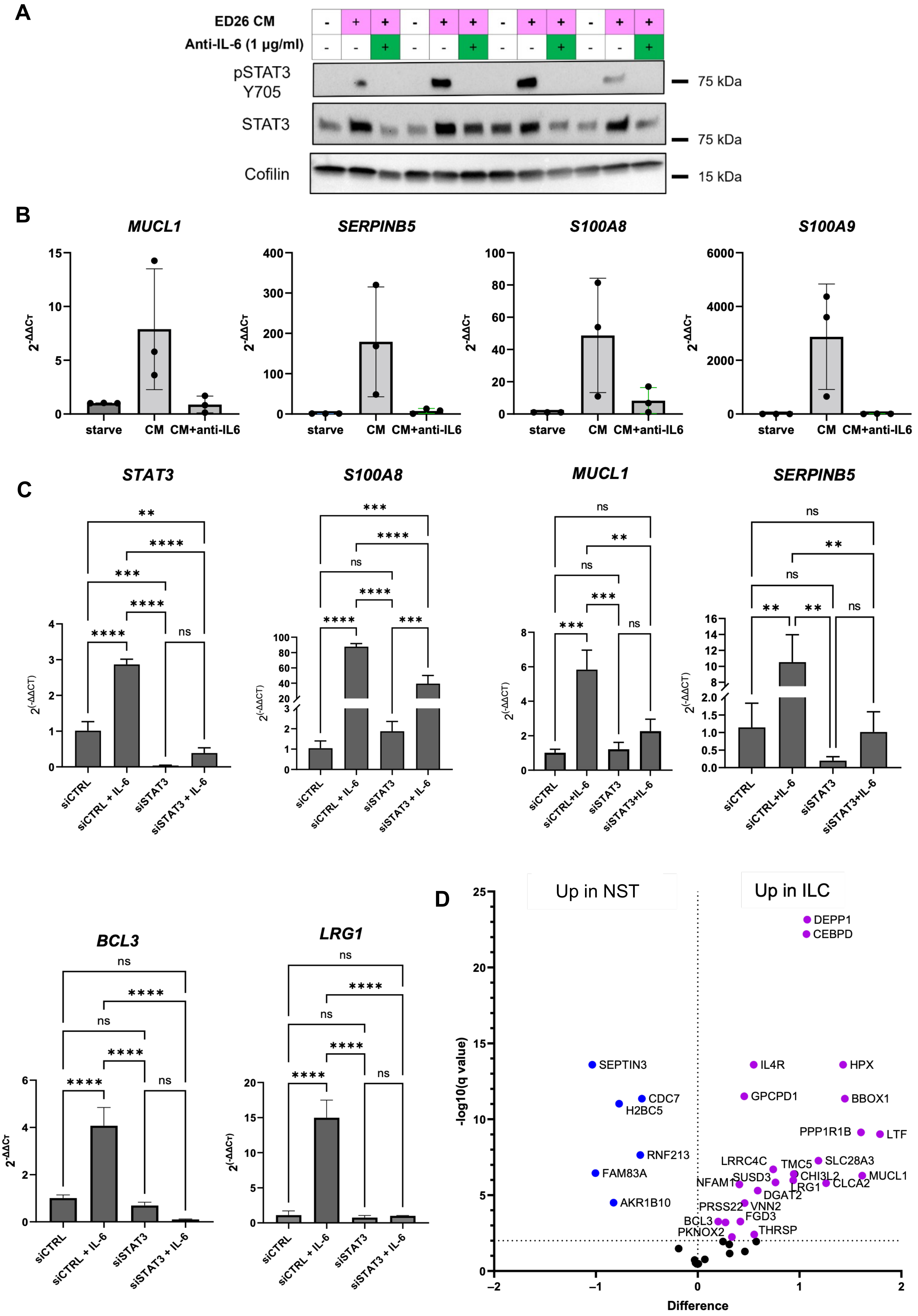

Supplementary Figure S4 RNA-Seq analysis of CAF conditioned media and IL-6 dependent gene changes in SUM44PE cells. A Western blot of SUM44PE cells stimulated with ILC CAF-CM +/- anti-IL6 (1 µg/mL) for 24 hours. **B** RT-qPCR validation of significant genes detected in RNA-Seq analysis in SUM44PE cells stimulated with ILC CAF-CM +/- anti-IL6 for 24 hours, n=3 biological replicates. **C** RT-qPCR validation of significant genes in RNA-Seq analysis in SUM44PE cells treated with siCTRL or siSTAT3 +/- IL-6 for 24 hours, n=3. One-way ANOVA with Tukey's multiple comparison test in Graphpad Prism, ** adjusted p-value <0.01, *** p<0.001, **** p<0.0001. **D** Volcano plot of IL6 gene signature genes in ER+ ILC (n=191) compared to ER+ NST (n=555) tumors in the TCGA RNA-Seq dataset. Multiple unpaired t-tests with BKY correction in GraphPad Prism, FDR<0.05 for significance.

Supplementary Figure S5

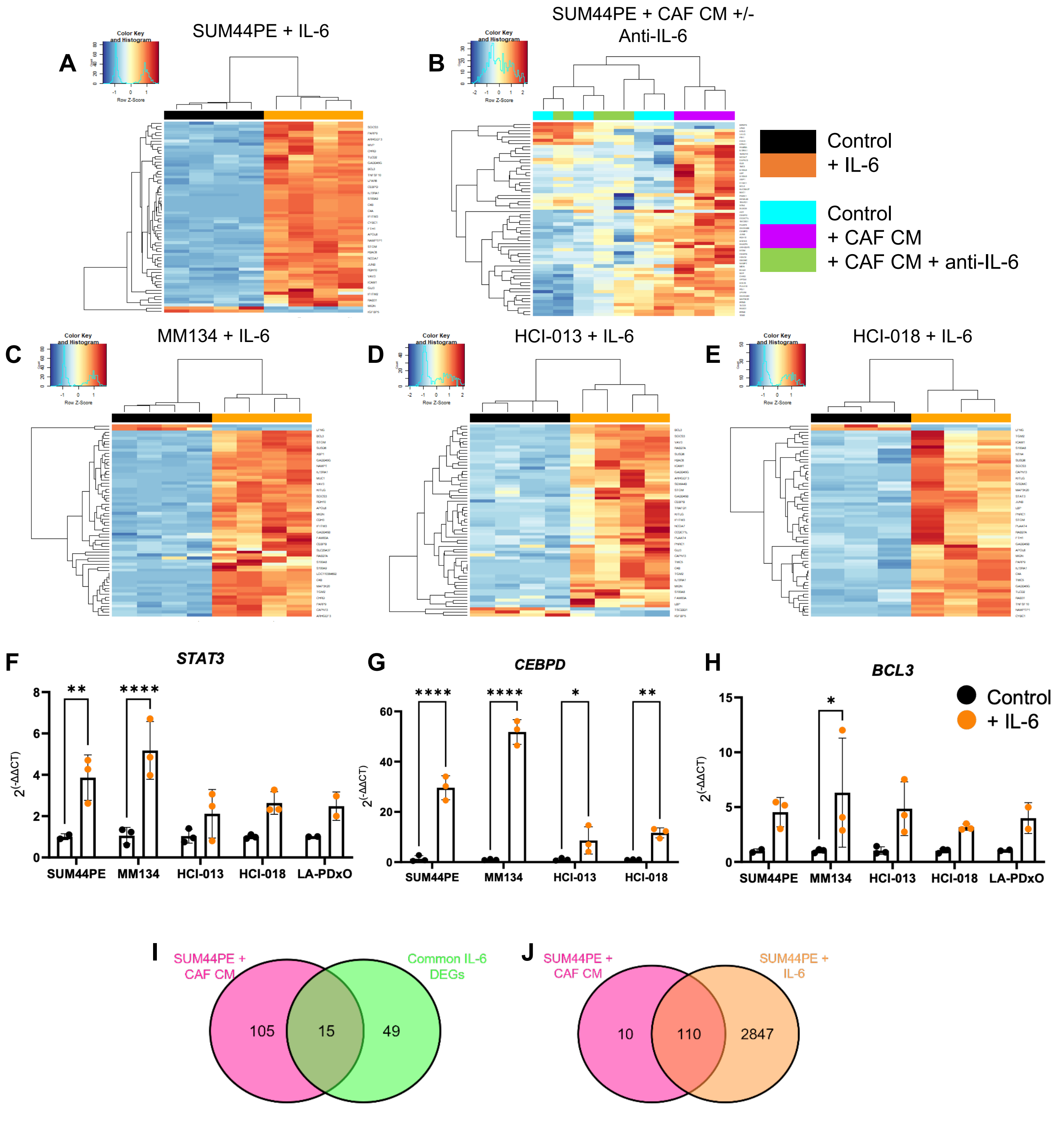

Supplementary Figure S5 RNA-Seq analysis of IL-6 dependent gene changes in ILC models. A-E Heatmaps of the consensus IL6GS genes in (A) SUM44PE cells + IL-6, (B) SUM44PE cells + CAF CM +/- anti-IL-6, (C) MM134 + IL-6, (D) HCI-013 + IL-6, and (E) HCI-018 + IL-6 for 24 hours. **F-H** qPCR validation of consensus IL6GS genes (F) *STAT3,* (G) *CEBPD* and (H) *BCL3* in SUM44PE, MM134, HCI-013, HCI-018 and LA-PDxO ILC models (n=3 biological replicates, except for LA-PDxO, n=2), two-way ANOVA with Šídák's multiple comparisons test, * adjusted p-value<0.05, ** p<0.01, *** p<0.001, ****<0.0001. Venn diagrams showing overlap between the 110 CAF CM induced genes in SUM44PE cells with **I** the consensus IL6GS and **J** the genes induced by IL-6 in SUM44PE cells.

Supplementary Figure S6

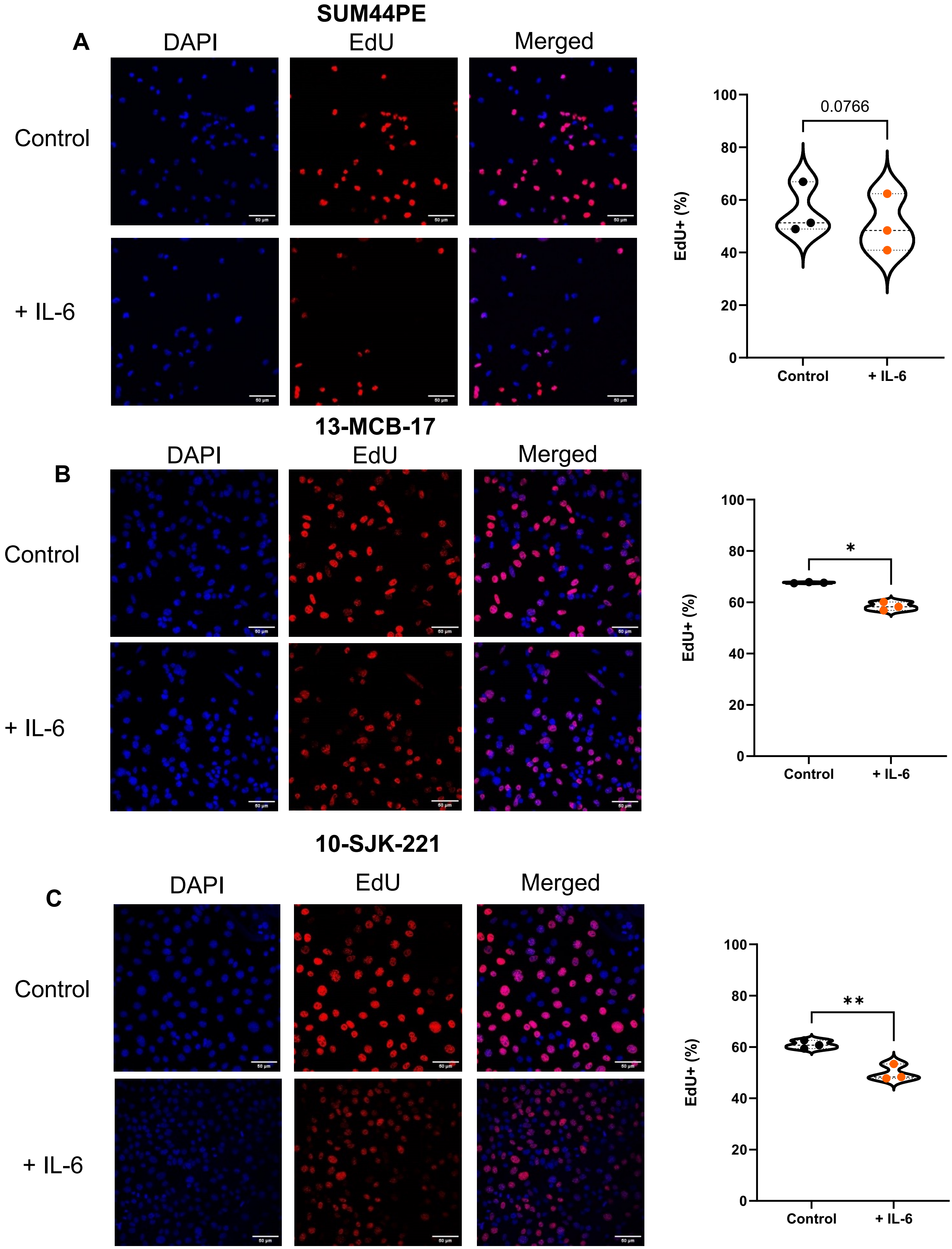

Supplementary Figure S6 IL-6 inhibits ILC proliferation. A Representative images of SUM44PE cells in growth factor depleted media (control) or +IL-6 for 24 hours, with nuclei labelled with DAPI (blue) and EdU+ nuclei labelled with TAMRA-azide (red), scale bar 50 μm. Right - quantification of mean % of EdU+ cells three independent repeats. **B, C** Representative images for untreated and 20 ng/ml recombinant mIL-6 treated (24 hours) 13-MCB-17 and 10-SJK-221 mILC cells with nuclei labelled with DAPI (blue) and EdU+ nuclei labelled with TAMRA-azide (red). Graphs showing percentage of EdU positive 13-MCB-17 and 10-SJK-221 cells after 24 hours. Paired t-test in GraphPad Prism (*p< 0.05, **p < 0.01). Scale bar 50 μm, images taken at 40X on Olympus FV3000 microscope.

Supplementary Figure S7

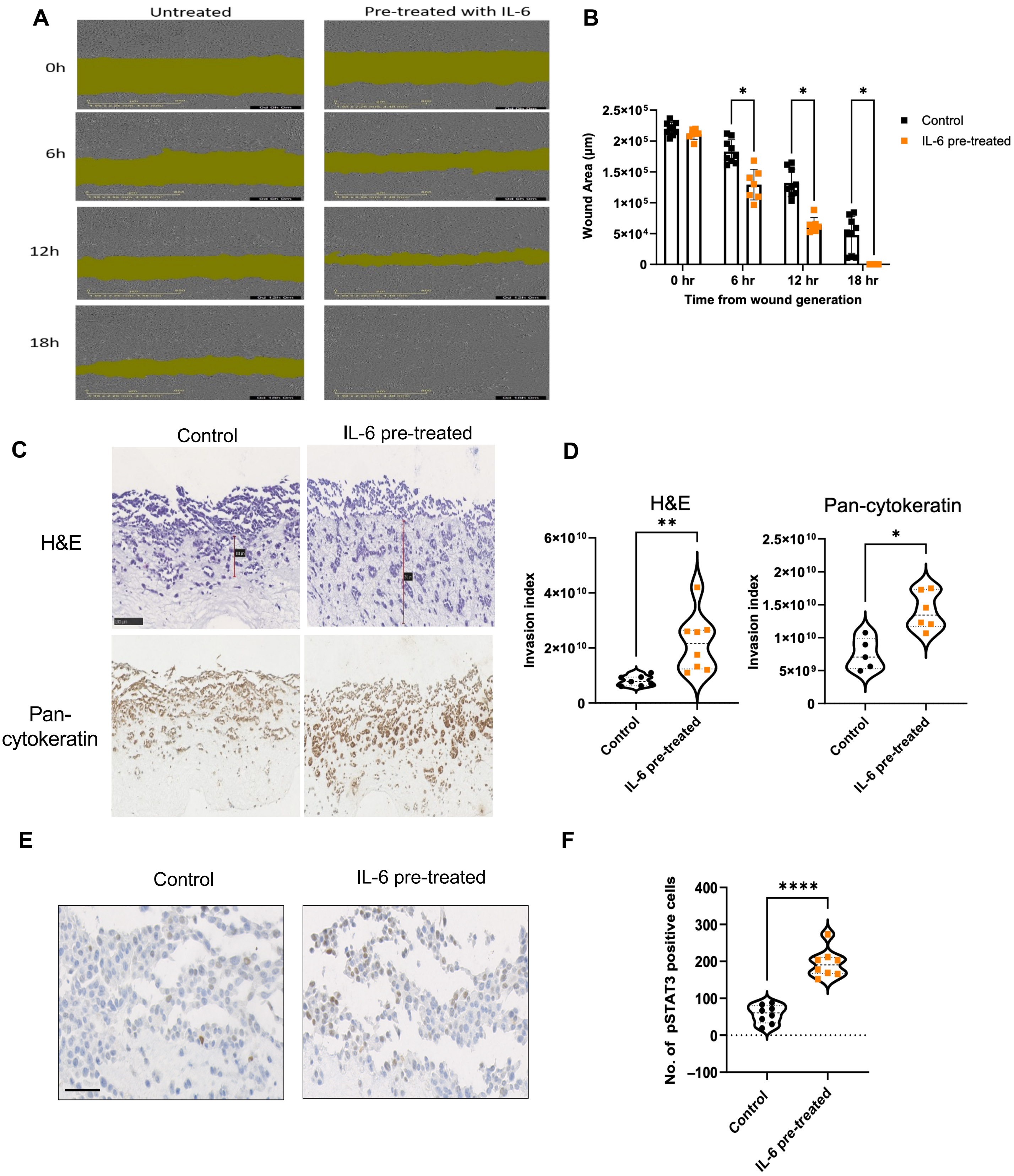

Supplementary Figure S7 IL-6 promotes migration and invasion of mILC cells. A Migration was assessed using a wound-healing assay where 13-MCB-17 cells were serum-starved and then pre-treated for 24 hours with or without recombinant mouse IL-6 (20 ng/mL). Representative images of wound closure were taken at 0 h, 6 h, 12 h, and 18 h. Scale bar, 600 µm. **B** Quantification of wound closure presented as the wound area in relation to the value of the initial wound generated, measured in ImageJ. Multiple unpaired t-tests in GraphPad Prism where * $p < 0.05$, ** $p < 0.01$, *** $p < 0.\ 001$, **** $p < 0.0001$. **C** Representative images of H&E (top) and pan-cytokeratin (bottom) stained sections showing 13-MCB-17 cell cultured on top of collagen/fibroblast (CAF 9188) matrices at day 5 of the organotypic invasion assay, pre-treated with or without IL-6 for 24 hours before seeding. Images captured using Hamamatsu Nanozoomer XR, scale bar 100 µm. **D** Quantification of the invasion index of 13-MCB-17 cells based on H&E staining (left) and pan-cytokeratin staining (right), paired t-tests in Graphpad Prism, * $p<0.05$, ** $p<0.01$. **E** Representative images of STAT3 pY705 staining in organotypic sections, scale bar 25 µm. **F** Quantification of the number of pSTAT3+ cells detected in 1.5×10^5 μm^2 area regions, unpaired t-test in Graphpad Prism, **** $p<0.0001$.

Supplementary Figure S8

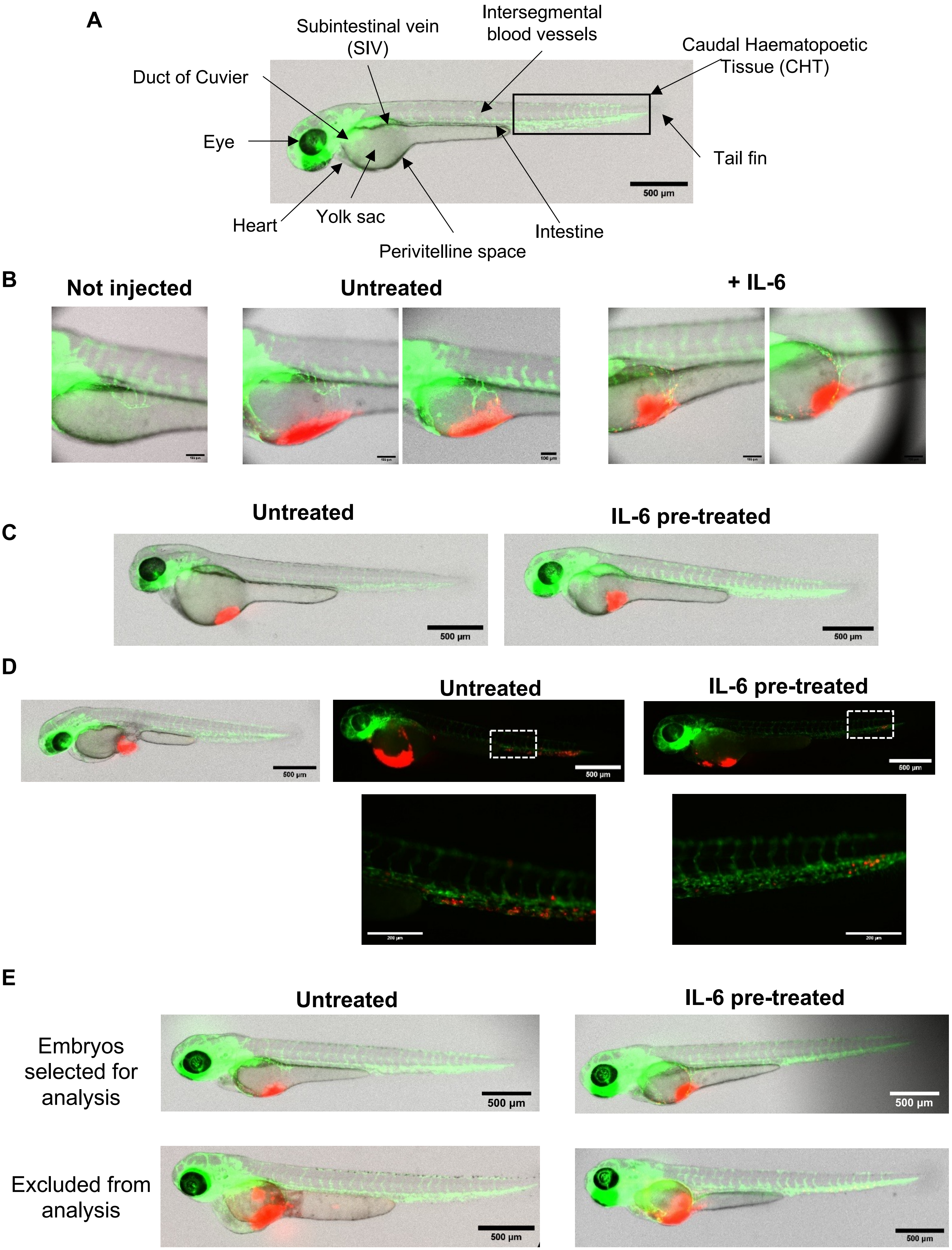

Fli1:eGFP labelled zebrafish vasculature; SUM44PE tumour cells

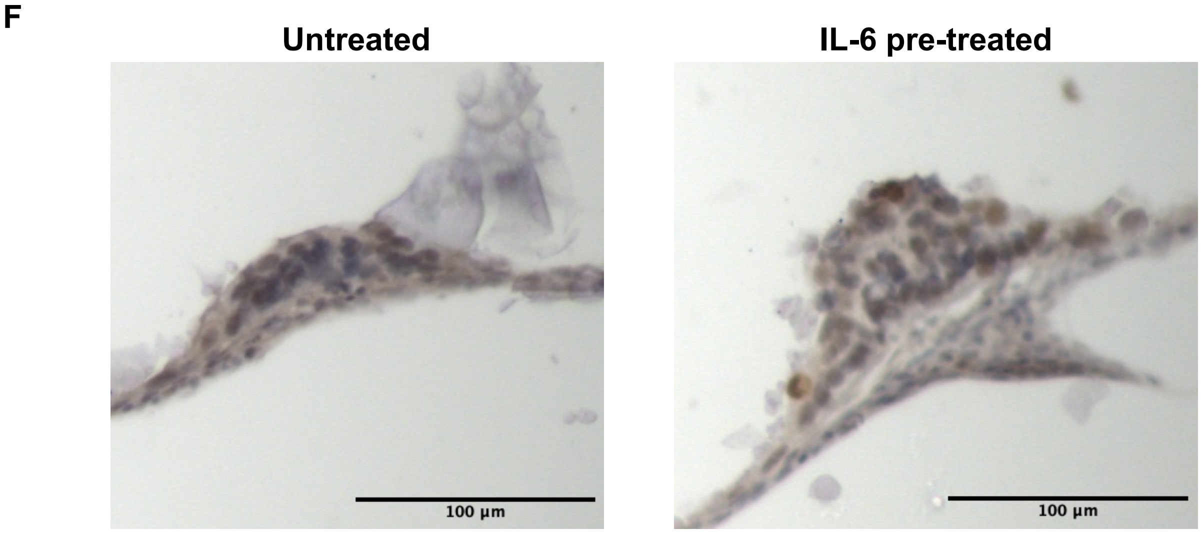

Supplementary Figure S8 Zebrafish xenograft assay A Image of uninjected 2dpf Casper *Tg(fli1*:eGFP) embryo. **B** Representative images of of 3dpf/1days post injection *Tg(fli1*:eGFP) embryo, left to right – not injected, injected with untreated SUM44PE cells, injected with IL-6 pre-treated SUM44PE cells. Images taken at 16x on a mesoscope, scale bar shows 100 μm. **C** Representative images of correctly injected 2dpf embryos 2-4 hours post injection kept for remainder of experiment, left – injected with untreated SUM44PE and right – IL-6 pre-treated SUM44PE cells. **D** Examples of rejected embryos at 2dpf: left – yolk sac defect and cardiac oedema, centre and right – embryos with SUM44PE cells injected directly into the circulation. White boxes and insets show disseminated cells in the CHT at 16x. **E** Embryos at 3dpf/1dpi. Top – embryos selected for continued analysis, bottom – embryos with very large tumor masses causing cardiac oedema and yolk defects, excluded from further analysis. (C-E) Images on mesoscope at 3.2x, scale bar shows 500 μm. *fli1*:eGFP labelled vasculature in green, DiI-dyed SUM44PE cells in red. **F** Immunohistochemical staining of STAT3 in (left) untreated and (right) IL-6 pre-treated SUM44PE tumors in the PVS injection site 4dpf/2dpi embryos, scale bars show 100 μm.

Supplementary Figure S9

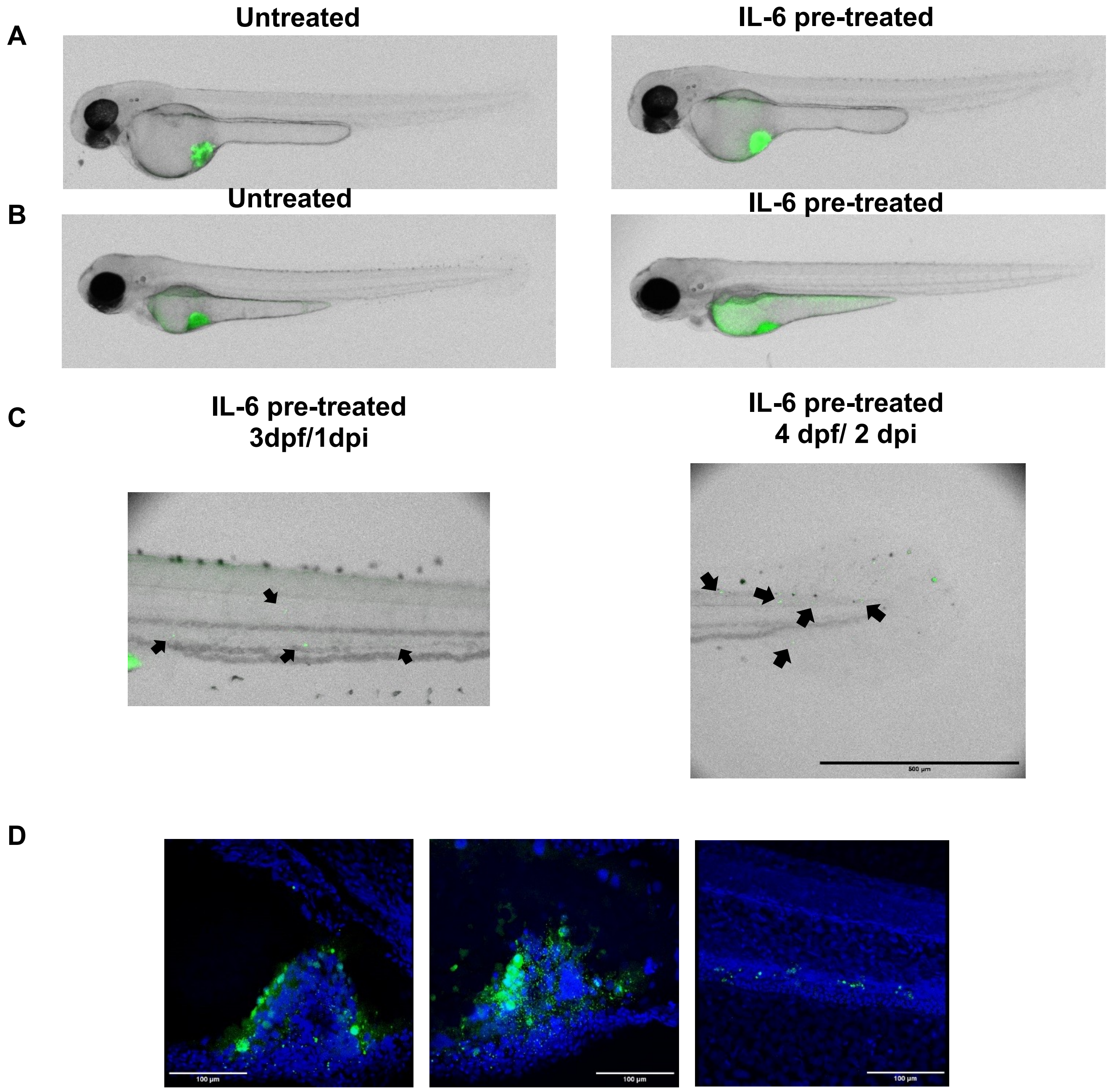

Supplementary Figure S9 Zebrafish xenograft assay A 2dpf Casper zebrafish embryos 2-4 hours after injection with SUM44PE Luc-zsGreen cells untreated (left) or pre-treated with IL-6 (right). **B** Embryos at 3dpf/1dpi after injection with SUM44PE Luc-zsGreen cells untreated (left) or pre-treated with IL-6 (right). **C** Evidence of disseminated cells in embryos injected with IL-6 pre-treated cells at 3dpf/1dpi (left) and 4dpf/2dpi (right). **D** 3D-projections of fixed embryos at 2 dpi injected with IL-6 pre-treated SUM44PE Luc-ZsGreen cells (green) in the embryo PVS injection site in the yolk sac (left and middle) and in the caudal haematopoietic tissue, nuclei labelled with Hoechst (blue).

Supplementary – raw western blots

Figure 1E

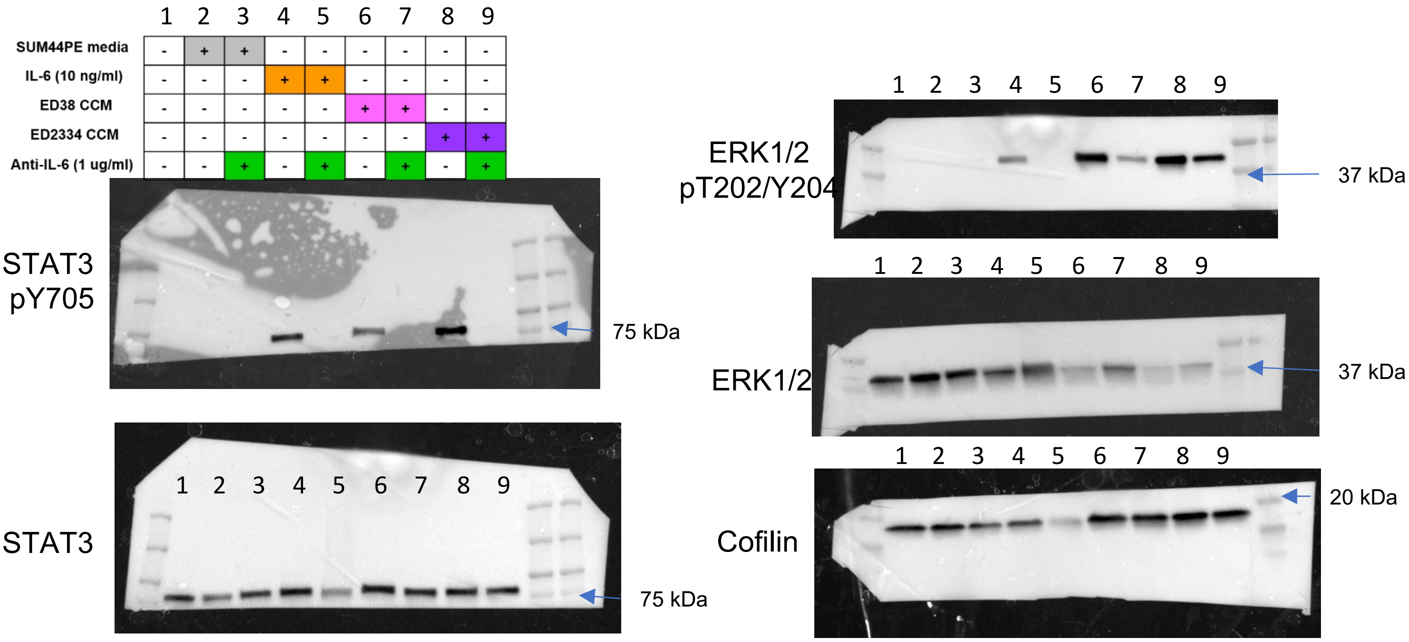

Figure 6D

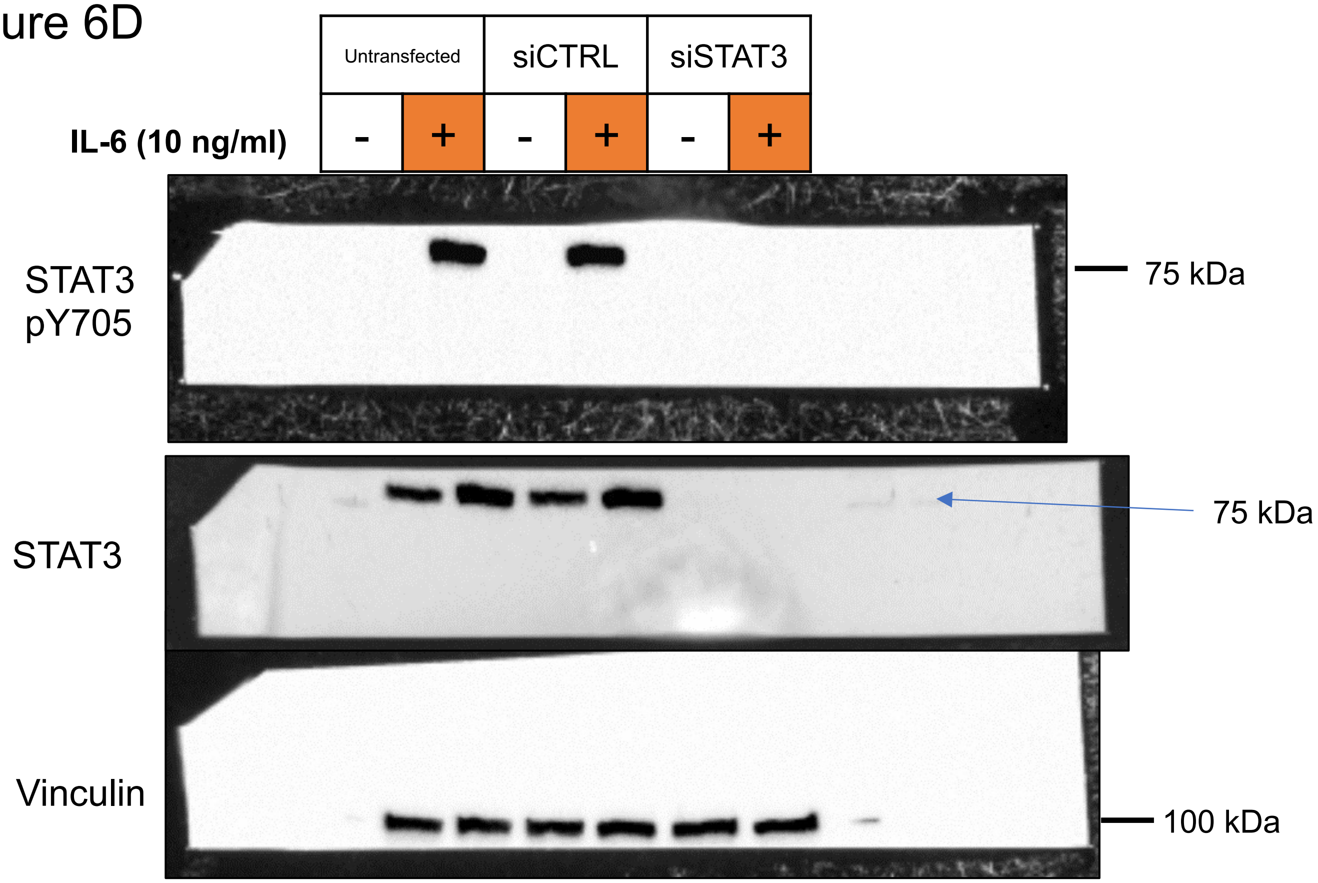

Supplementary Figure S1

S1A

Repeat 1			Repeat 2			Repeat 3			Repeat 4		
Control	30 mins	24 hrs	Control	30 mins	24 hrs	Control	30 mins	24 hrs	Control	30 mins	24 hrs

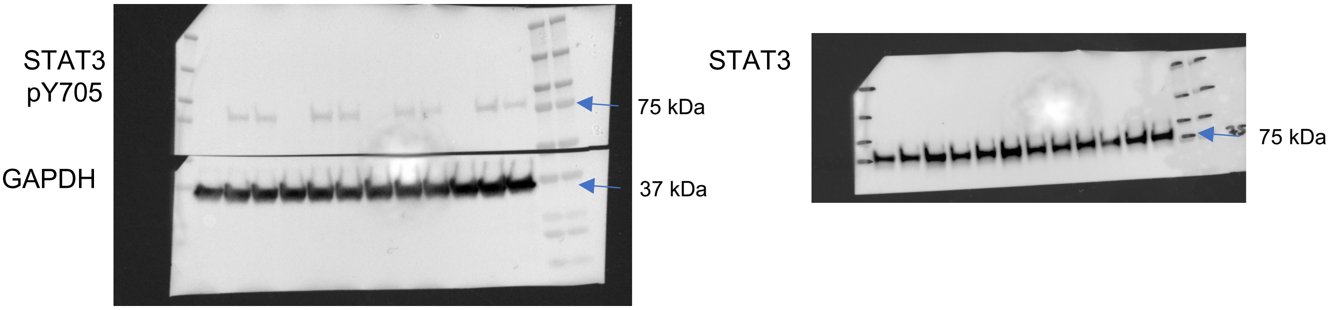

S1B

30 min					24 hours				
0	0.1	1	10	100	0	0.1	1	10	100
1	2	3	4	5	6	7	8	9	10

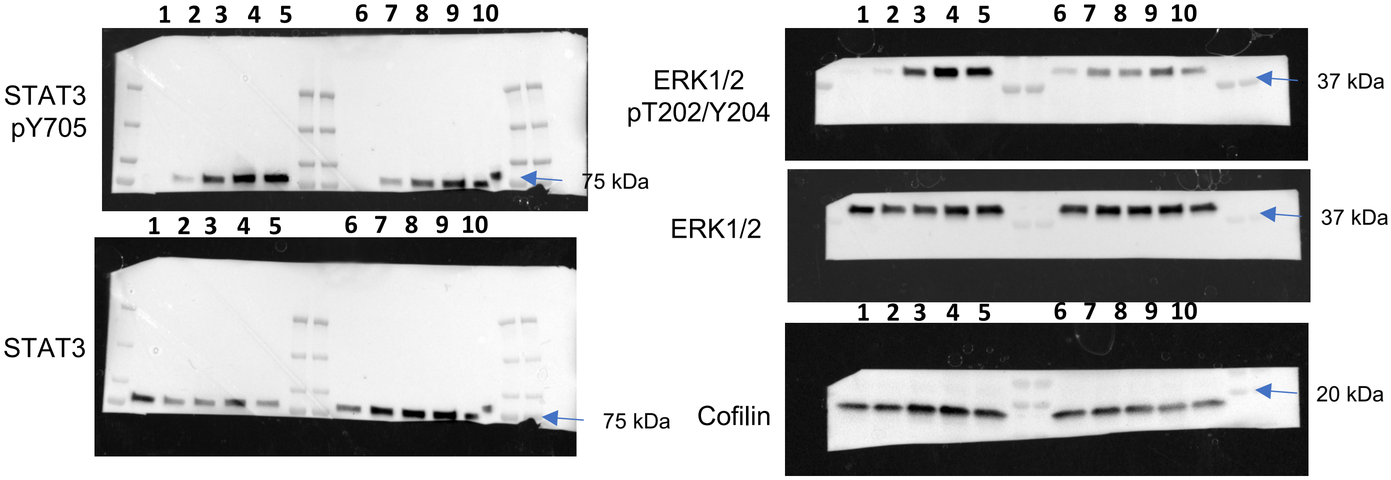

S1G

	1	2	3	4	5
IL-6 (10 ng/ml)	-	+	+	-	-
ED26 CAF CM	-	-	-	+	+
Anti-IL-6 (1 μg/ml)	-	-	+	-	+

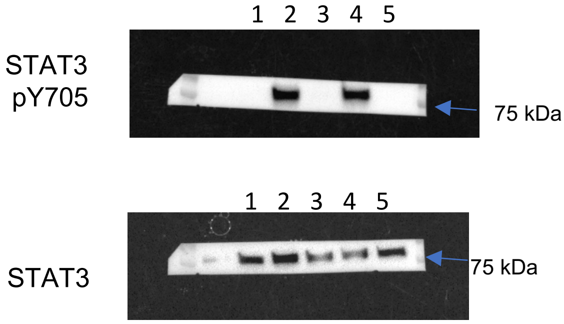

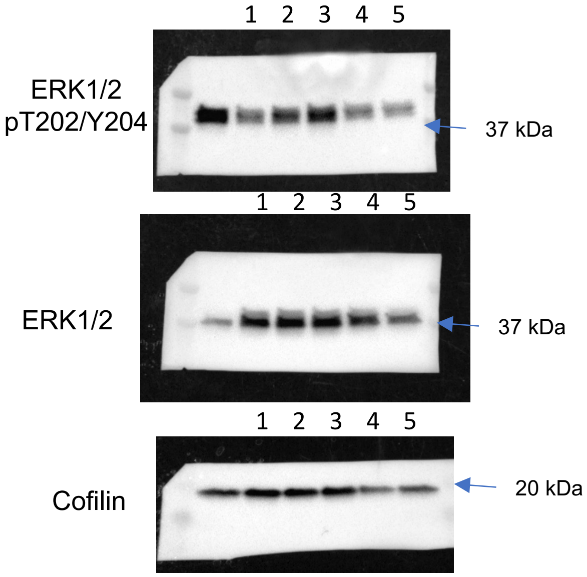

S1H

30 min

	13-MCB-17						10-SJK-221					
WEPtn media	-	+	-	-	-	-	-	+	-	-	-	-
mIL-6 (20 ng.ml)	-	-	+	+	-	-	-	-	+	+	-	-
CAF 9188 CM	-	-	-	-	+	+	-	-	-	-	+	+
Anti-IL-6 (1 µg/ml)	-	-	-	+	-	+	-	-	-	+	-	+

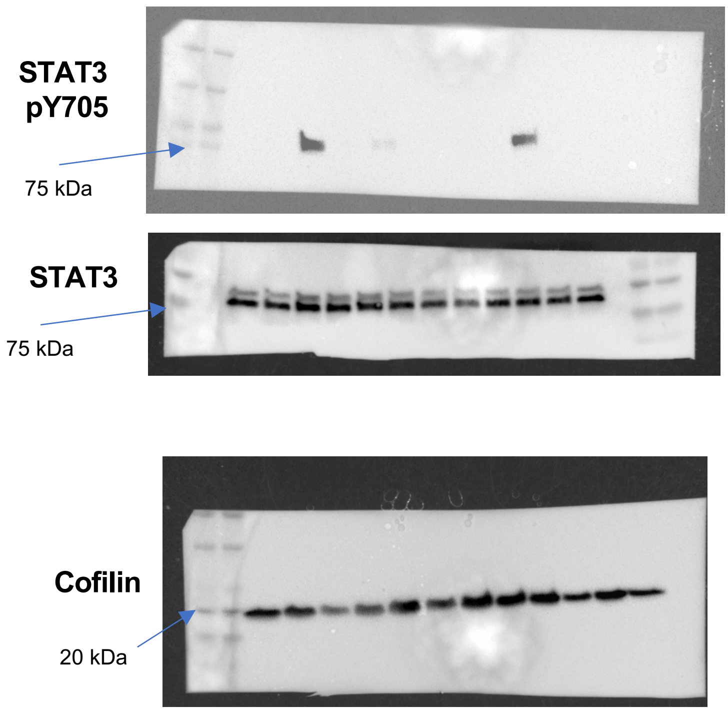

24 hr

	13-MCB-17						10-SJK-221					
WEPtn media	-	+	-	-	-	-	-	+	-	-	-	-
mIL-6 (20 ng.ml)	-	-	+	+	-	-	-	-	+	+	-	-
CAF 9188 CM	-	-	-	-	+	+	-	-	-	-	+	+
Anti-IL-6 (1 µg/ml)	-	-	-	+	-	+	-	-	-	+	-	+

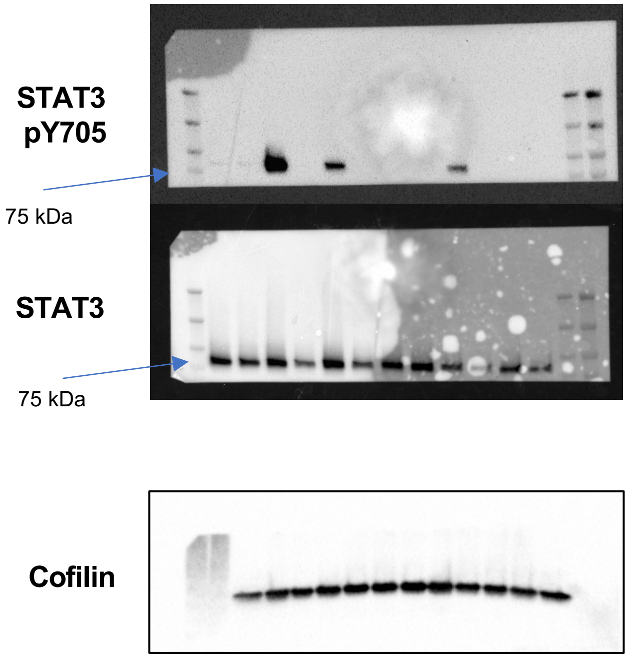

30 min

S1I - left

IL-6 (10 ng/ml)	-	-	-	+	+	+	-	-	-
ED38 CAF CM	-	-	-	-	-	-	+	+	+
Anti-IL-6 (1 µg/ml)	-	+	-	-	+	-	-	+	-
Baricitinib (125 nM)	-	-	+	-	-	+	-	-	+

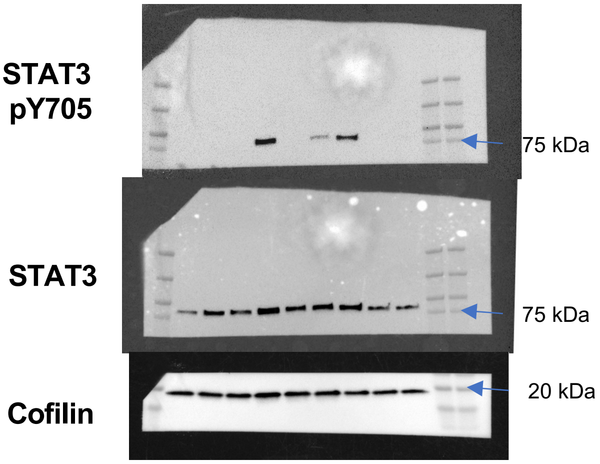

S1I - right

24 hr

IL-6 (10 ng/ml)	-	-	-	+	+	-	+	-	-
ED38 CAF CM	-	-	-	-	-	+	-	+	+
Anti-IL-6 (1 µg/ml)	-	+	-	-	+	-	-	+	-
Baricitinib (125 nM)	-	-	+	-	-	-	+	-	+

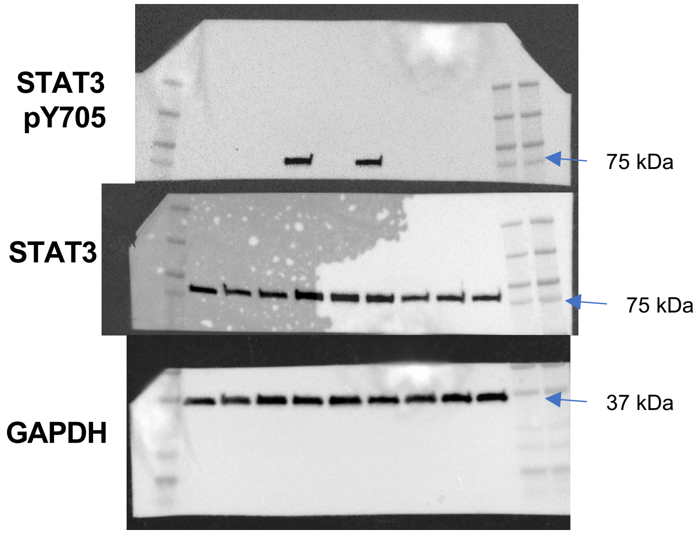

Supplementary Figure S4A

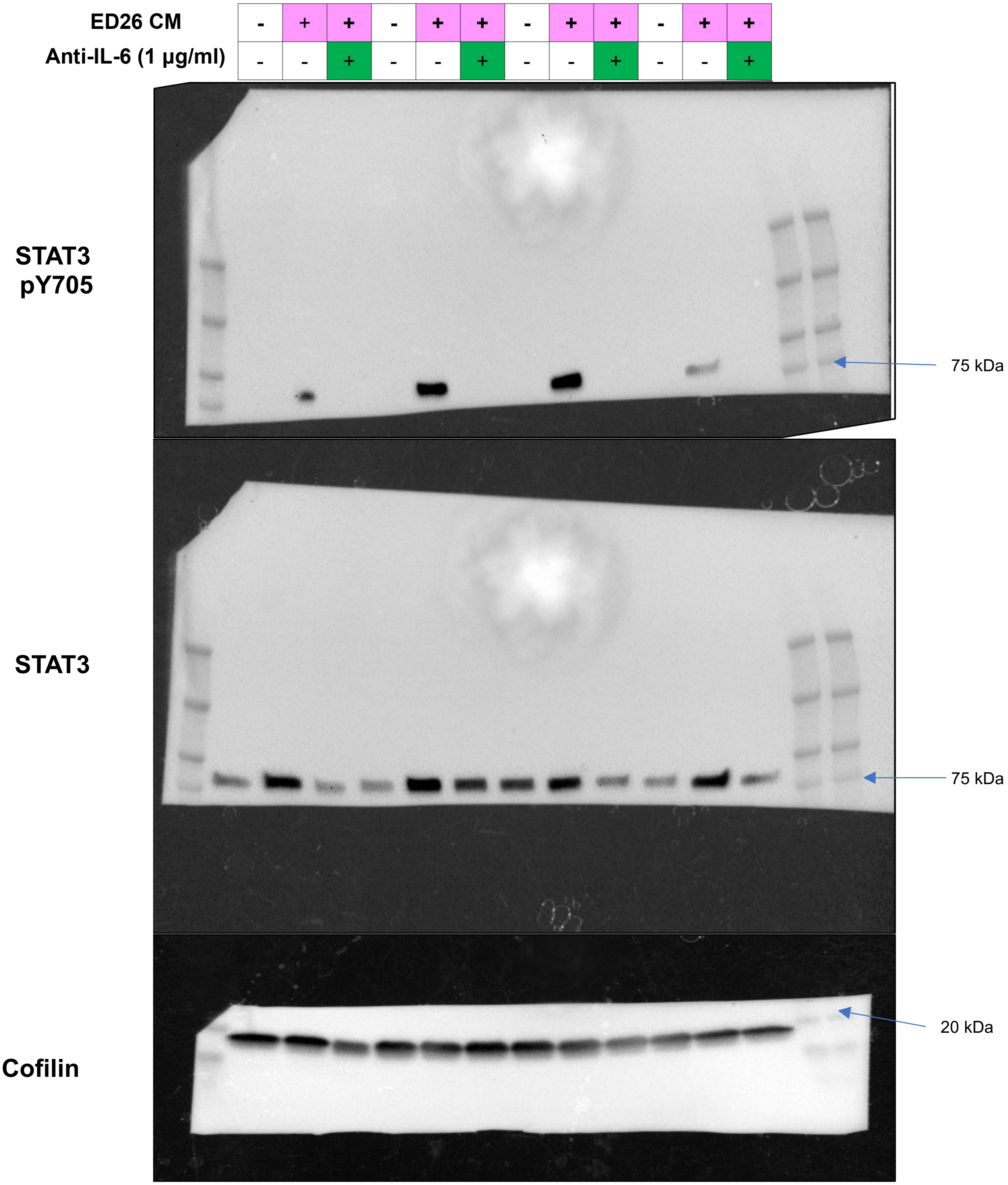

Supplement: Supplementary file 1 — Supplementary Material 1 [file 13058_2025_2074_MOESM1_ESM.pdf]
